# Micro- and nano-plastics induce inflammation and cell death in human cells

**DOI:** 10.3389/fimmu.2025.1528502

**Published:** 2025-03-31

**Authors:** Brandon Bishop, William S. Webber, Shaikh M. Atif, Ashley Ley, Karl A. Pankratz, Rachael Kostelecky, Sean P. Colgan, Charles A. Dinarello, Wei Zhang, Suzhao Li

**Affiliations:** ^1^ Department of Chemistry, University of Colorado Boulder, Boulder, CO, United States; ^2^ Department of Medicine, University of Colorado Denver Anschutz Medical Campus, Aurora, CO, United States; ^3^ Division of Allergy and Clinical Immunology, Department of Medicine, University of Colorado Denver Anschutz Medical Campus, Aurora, CO, United States; ^4^ Mucosal Inflammation Program, Division of Gastroenterology and Hepatology, Department of Medicine, University of Colorado Anschutz Medical Campus, Aurora, CO, United States

**Keywords:** micro- and nano- plastics (MNPLs), inflammation, cell death, human, environment, health

## Abstract

**Introduction:**

The presence of micro- and nano-plastics (MNPLs) in the environment has increased significantly in the past decades. However, the direct impact of MNPL particles on human health remains unclear.

**Methods:**

In this study, we utilized a modified extraction method with a previously reported staining technique to develop a novel approach for identifying individual plastics in mixtures of MNPLs of commercial and environmental origins to be able to investigate their impacts on human cell inflammation and cell death. Polypropylene (PP), polyethylene (PE), polystyrene (PS), and polyethylene terephthalate (PET) were the plastics analyzed. The plastic composition of the environmental MNPLs was characterized using multiple analytical techniques, including Fourier transform infrared spectroscopy, confocal imaging, scanning electron microscopy, and X-ray diffraction.

**Results:**

We found that both commercial and environmental MNPLs, especially PET, impose a strong inflammatory response on various human cells and tissues. At 1 mg/mL, they robustly stimulate inflammatory IL-1β and IL-6 secretion in a time-dependent manner. Importantly, we observed that the MNPLs induced variable inflammatory responses in cells depending on their plastic composition. Environmental samples rich in PET showed a strong dose-dependent response and induced IL-1β secretion at doses as low as 100 ng/mL. In addition, MNPLs can induce human cell death with or without obviously altering the cell morphology.

**Discussion:**

These findings are significant because they represent the first instance of authentic MNPLs being collected from ecological water samples for characterization and the first time the direct influences of commercial and environmental MNPLs have been compared in human cell studies. The methods developed in this study provide a foundation for future research to isolate MNPLs from the environment and explore their potential impacts on human health and disease development.

## Introduction

Plastics are synthetic polymers present in nearly all man-made items ranging from beverage bottles and food wraps to electronics, cosmetics, and automobiles. Almost every facet of our lives has been touched by plastics. Global production of polymer resins and fibers reached 380 million metric tons in 2015 (1, 2). Less than 10% of these polymers had been recycled as of 2015, and ~79% of them, over 6,000 million metric tons, ended up in landfills (3). During a potentially centuries-long afterlife as trash, this polymeric debris is slowly degraded into microplastics (MPs, 1 μm to 5 mm) and nanoplastics (NPs, 1 nm to 1 μm) through mechanical weathering, photodegradation, and microbial activities, and eventually released into the environment through natural weather processes and human activities (4). Micro- and nano- plastics (MNPLs) are the most numerically abundant plastic debris found in our environment (5). A large portion of these MNPLs ultimately find their way into drinking water sources and waterways used by humans for commercial and recreational purposes (4). It has been estimated that 74,000 to 121,000 MNPL particles are consumed annually by individuals in the United States through the digestive and respiratory systems with recent studies reporting significant amounts of MNPL particles in the blood and feces of healthy Americans (6–8). As the plastic problem continues to escalate, there is an urgent need to determine the impacts of MNPLs on human health.

Chronic exposure to environmental xenobiotics and pollutants has been found to result in systemic inflammation and chronic disease in living organisms (9–16). In animal species, reduced fecundity in copepods and developmental effects in urchin embryos exposed to MNPLs have been reported, as well as metabolic shifts toward glycolysis, reduced mitochondrial respiration and intestinal inflammation in rodents (17–20). In humans, MNPLs have been detected in a wide range of biological samples, tissues and organs, including feces, urine, blood, placenta, lung, intestines and liver (6, 21–26). Moreover, significant amounts of MNPL particles have been detected in people with inflammatory bowel disease (IBD), liver cirrhosis, and cardiovascular disease (7, 25, 27). Importantly, recent research found greater incidence of myocardial infarction, stroke, and death among patients undergoing carotid endarterectomy for high-grade carotid artery stenosis when MNPLs were identified within the atheroma (27). Together, these findings suggest that polymeric contaminants from food and water accrue in living tissue/organs and are associated with disease. However, it remains unknown whether MNPL exposure directly causes inflammation or disease.

An important goal of our study is to distinguish the health effects caused by exposure to commercial MNPLs versus environmental MNPLs, as microplastics exposed to the environment can adsorb and concentrate harmful pollutants (28–33). Studies have shown that the concentration of contaminants adsorbed onto a polymer is 10^6^ times greater relative to the amount in solution (34). Moreover, it is speculated that environmental MNPLs can act as a vector and facilitate the entrance of toxic materials into living organisms through a cellular interaction acting as a “bridge” or “carrier” (28, 32). In the present study, we developed a novel approach for identifying individual plastics in mixtures of MNPLs obtained from commercial sources and environmental MPs obtained from natural water resources by combining a modified version of an established extraction method with a previously reported staining technique for laboratory plastics. The impacts of the commercial and environmental MNPLs on inflammation, cell morphology and survival were studied in parallel in human cell cultures.

Four types of major plastics – polypropylene (PP), polyethylene (PE), polystyrene (PS), and polyethylene terephthalate (PET) – were prepared or isolated from commercial and environmental water samples, respectively, and applied to human cell cultures. The MPs in major creeks and lakes that provide primary water sources for the Greater Denver Area region were investigated. Special attention was paid to locations where wastewater reclamation and sewage treatment plants are located. These locations were chosen based on accessibility, economic viability, and designation as residential versus commercial water sources. Boulder Creek and Boulder Reservoir are residential water sources. However, Boulder Creek has city run-off directed into it. South Platte River is a commercial water source with industrial and agricultural run-off. Marshall Lake is a recreational water source recently exposed to a large-scale wildfire, chosen to examine the impact of environmental disasters on the amount of plastics in bodies of water. The total amount of MPs, particle size, and plastic composition were examined for each location. Commercially available synthetically pure plastics were used for comparison. The commercial MNPL samples of each plastic type were sized to be < 400 nm or between the range of 400 nm-300 μm. This provided pure commercial MNPLs in both the nano and micro size ranges. The stability of the particles in solution and the upper size limit was confirmed by dynamic light scattering (DLS). The size range for DLS is 0.3 nm-10 μm (35–37). As such, the larger MNPL particles were imaged using scanning electron microscopy (SEM) to confirm their size. The structural integrity of the commercial polymer samples was analyzed using X-ray diffraction (XRD). The health effects of the MNPLs were studied in various human cell cultures, using both commercial and environmental samples. Their effects on human cell inflammation, cell morphology, cell apoptosis, and necrosis were investigated. We found that the environmental plastic samples induced variable inflammatory responses in cells depending on their plastic composition. PET, among the different types of plastics tested, exerted the strongest inflammatory responses in human cell and whole blood cultures. Both commercial PET and environmental plastic samples rich in PET induced cell death. Further, we observed that environmental MPs rich in PET induce more robust inflammatory responses, and more significant cell damage effects on human cell morphology and survival than commercial PET alone. In summary, with the new methods developed in this study our findings demonstrate that direct exposure to MNPLs has detrimental effects on human cells, and future studies are desired to investigate the impacts of chronic MNPL exposure on human health and disease development.

## Results

### Characterization of commercial MNPLs from commercial resources

**Figure 1:**
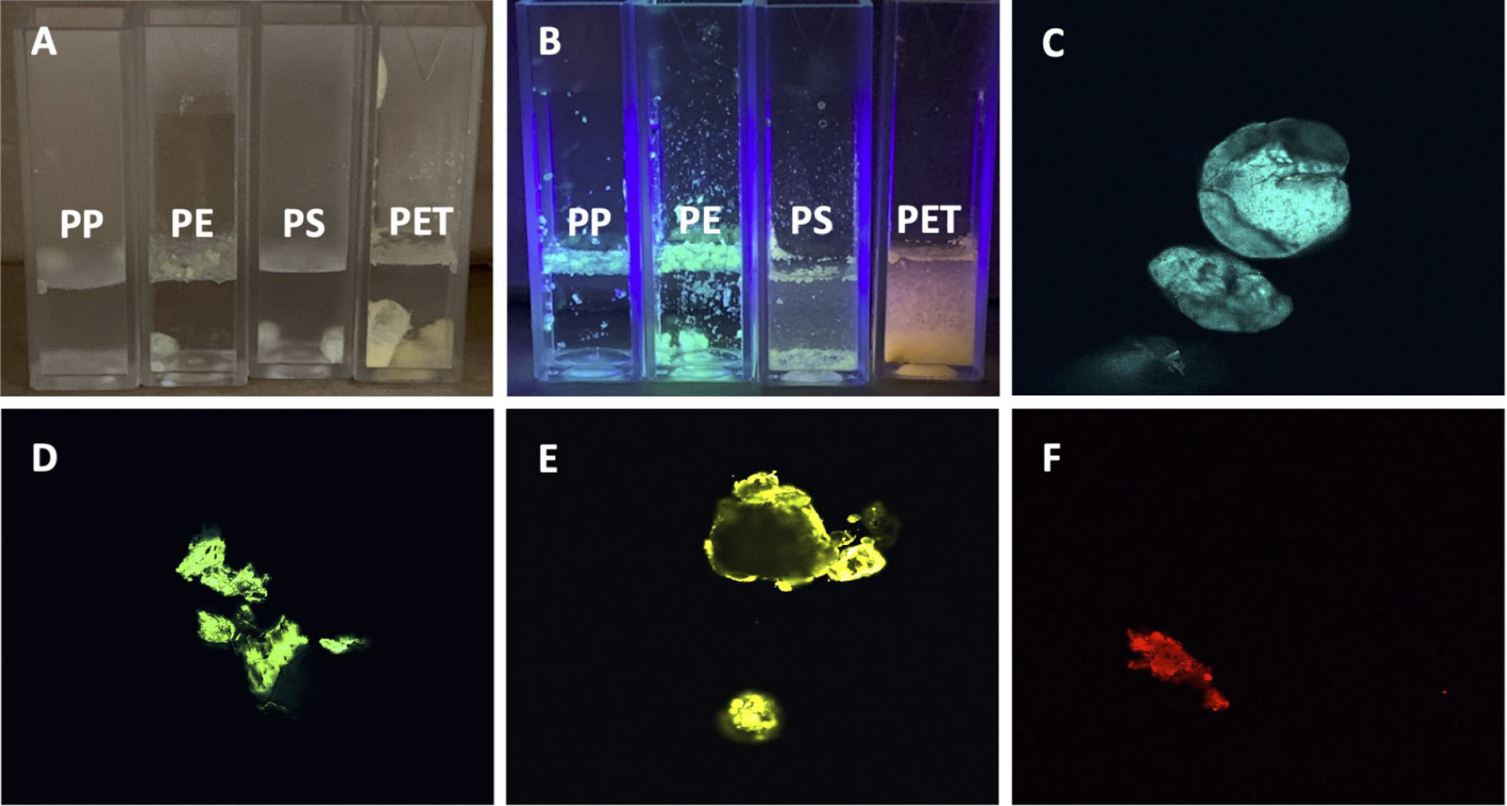
Fluorescent images of DANS-stained commercial MNPLs in DI water. **(A)** Image of commercial MNPLs stained using 4-dimethylamino-4’-nitrostilbene (DANS) fluorescent dye suspended in DI water, no UV illumination. **(B)** Image of stained commercial MNLPs using DANS fluorescent dye suspended in DI water, illuminated by UV LED. **(C–F)** Fluorescent confocal microscopy images of stained commercial plastics. **(C)** for PP, **(D)** for LDPE, **(E)** for PS, **(F)** for PET.

To establish a standard method for identification of MNPLs from environmental water sources, we first generated MNPLs of PP, PE, PS, and PET origin using commercial resources. FTIR analysis was then conducted on the individual plastics. As expected, the FTIR analysis of the generated commercial MNPLs was consistent with previous literature, displaying the characteristic major peaks for each commercial polymer (**Supplementary Figure 1**) (38–40). Specifically, PE showed the four FTIR signals expected with the CH_2_ stretching frequencies showing up at 2915 cm^-1^ and 2850 cm^-1^. Furthermore, the fingerprint region shows the CH_2_ scissoring at 1465 cm^-1^ and the CH_2_ rocking at 720 cm^-1^. PP showed six FTIR signals as expected with the CH_2_ stretching frequencies showing up at 2915 cm^-1^, and 2840 cm^-1^. The CH_3_ stretching peaks are visible at 2950 cm^-1^, and 2855 cm^-1^. The fingerprint region shows the CH_2_ scissoring at 1465 cm^-1^ and the CH_3_ bending at 1375 cm^-1^. PET is easily distinguishable by FTIR due to the presence of the strong carbonyl peak at 1715 cm^-1^. The fingerprint region of PET shows the other two identifying peaks of the ester bond with the C-C-O stretch at 1230 cm^-1^ and the O-C-C stretch at 1095 cm^-1^. PET also shows the CH_2_ rocking peak at 720 cm^-1^. PS is also not difficult to identify from FTIR due to the aromatic CH stretching at 3025 cm^-1^. PS also shows CH_2_ stretching 2915 cm^-1^ and 2845 cm^-1^. The C-C stretching of the benzene ring is seen at 1600 cm^-1^. The fingerprint region of PS showed CH stretching of the aromatic ring at 1450 cm^-1^ and 1445 cm^-1^. The final dominating peak of PS is the ring bending peak at 690 cm^-1^. Distinguishing FTIR peaks of the individual plastics are given in **Table 1**. These dominating peaks were set as standards to analyze the plastic composition of the environmental samples and identify individual plastics. FTIR analysis can give a general idea of the polymers present in solution, however due to similarities in polymer backbones a more specific method of identification is necessary. Staining polymers with fluorescent dyes has recently become a popular method of identification due to the ability to image the polymers through fluorescent confocal microscopy (41–45). To confirm the polymers identified by FTIR, as well as establish a method of estimating the amount of individual commercial plastics in each environmental sample, we stained the generated MNPLs with 4-dimethylamino-4’-nitrostilbene (DANS) and imaged them using fluorescence confocal microscopy. As shown in **Figure 1**, each MP appeared as a specific color under UV illumination (**Figures 1A, B**) and could be identified using fluorescent confocal microscopy based on the differing fluorescence spectra after staining (**Figures 1C–F**). The fluorescence spectra for each polymer after staining matched what was previously reported. The polymers identified in the microscopy images were colored based on the color they appear under UV-light. The findings of PP: blue, PE: green, PS: yellow, and PET: red were consistent with what was previously reported (41).

**Table 1:** Major FTIR peaks for identification of standard commercial plastics.

Plastic	FTIR Peaks Used	Repeat Unit	Common Uses
Polystyrene (PS)	3025 cm^-1^, 690 cm^-1^	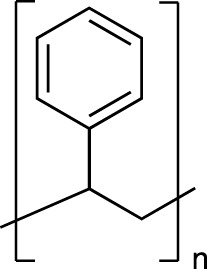	Styrofoam, foam packaging, food trays
Polyethylene (PE)	2850 cm^-1^	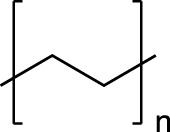	Plastic bags, water pipes and containers, Food containers, medical applications
Polypropylene (PP)	2950 cm^-1^, 1375 cm^-1^	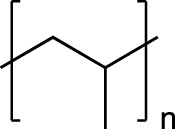	Plastic furniture, housewares, toys, automobile parts, pipes,
Polyethylene Terephthalate (PET)	1715 cm^-1^, 1095 cm^-1^	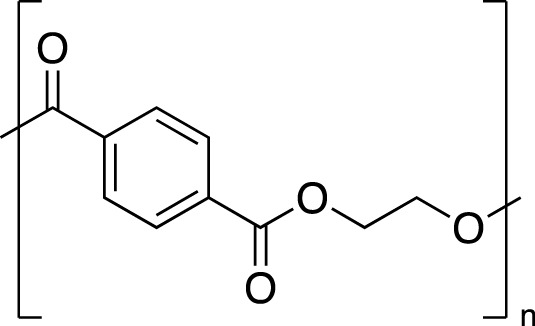	Single use water bottles, commercial beverage bottles, food and water containers, cosmetic products, sports products, cleaning products

The chemical structure, the standard dominating FTIR peaks used for identification in the environmental samples, and common uses of each standard plastic of PS, PE, PP and PET are listed.

**Figure 2:**
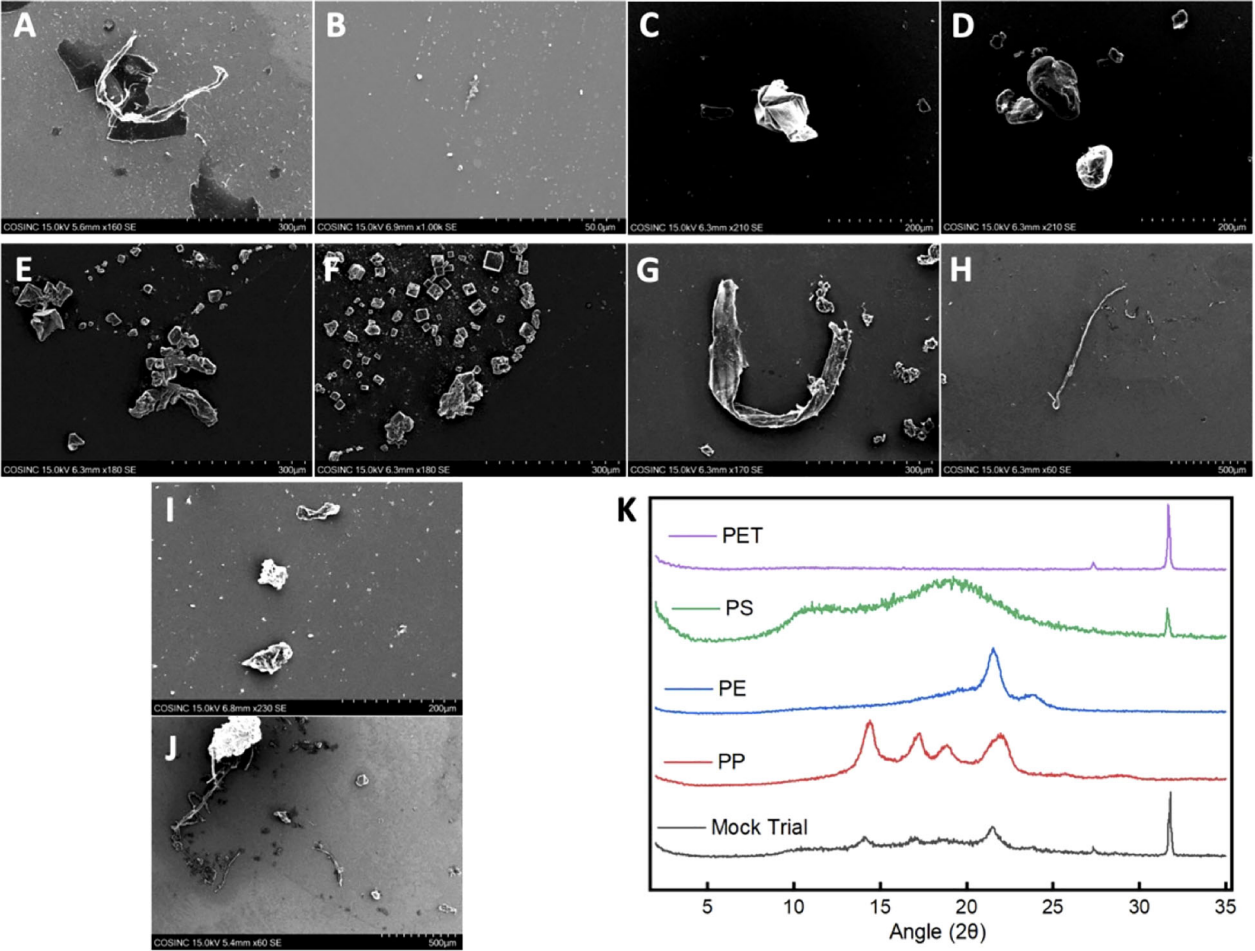
SEM images and XRD patterns of the commercial MNPLs and MPs obtained from the mock trial. **(A–J)** SEM Images of the commercial polymers and the MPs obtained from the mock trial: **(A, B)** SEM images of PP; **(C, D)** SEM images of PS; **(E, F)** SEM images of PET; **(G, H)** SEM images of PE; **(I, J)** SEM images of the mixture of MPs obtained from the mock trial after extraction. **(K)** Stacked XRD patterns of the commercial polymers and the mixture of MPs obtained from the mock trial after extraction.

It is well established that plastics have solubility issues and tend to coagulate and settle out of solution (36, 37). With this in mind, the commercial plastic solutions were also analyzed using DLS, SEM, and XRD. The commercial solutions with MNPLs that were < 0.4 μm in size were analyzed by DLS to confirm their size and examine their stability in solution. The DLS data revealed that the MNPL particles in solution were below the target size limit. Cumulant analysis was performed and the PDI reported was low (< 0.7) (**Supplementary Figure 2**). This indicates our size selection was effective and that the MNPL particles in solution are < 0.4 μm. The range of DLS is 0.3 nm-10 μm (37). This limited the number of MNPL solutions that could be analyzed by DLS. Solutions stored for extended periods of time (weeks to months) showed signs of coagulation, based on an increase in the PDI observed (**Supplementary Figures 2B, D, F, H**). However, with brief vortexing, DLS data revealed the MNPL particles in solution remained within the target size range. To avoid major coagulation and size issues, these solutions were used quickly and vortexed prior to use to disperse the plastics as evenly as possible and break up any coagulum. SEM imaging also showed that the plastics particles were under the desired size limit. SEM was taken on the plastic fractions that were < 300 μm. As shown in **Figure 2A–J**, the SEM of the commercial plastics displayed the expected polymeric structures: strand-like pieces were identified for PE and PP, cubic rod-shaped pieces were found for PET, and spherical particles were found for polystyrene. The XRD data for the commercial plastics provided spectrums identical to previous research (**Figure 2K**) (46–49). The commercial plastics used for this analysis were the same as the ones used for cell testing, confirming the structural integrity of the commercial samples used.

Next, a mock trial was conducted to verify that we could identify individual plastics in a mixture and to analyze the effectiveness of our extraction method. The MNPLs of PP, PE, PS, and PET obtained were mixed in DI water. The mixture was then subjected to the extraction procedure outlined in **Supplementary Figure 3**. A Fenton oxidation was performed followed by density separation using brine. The Fenton oxidation was chosen because it has been reported to be milder than regular hydrogen peroxide oxidation and other acidic and alkaline procedures (50–52). It has also been reported to be non-damaging to the four plastics being used in this study (50, 52). As shown in **Supplementary Figures 4A, B**, most of the MPs of mixed origins were captured in the top layer after density separation (~ 85% recovery). A small portion (~ 7.8%) was retained in the bottom inorganic sediment layer. The remaining 7.2% was lost during filtration and purification, which is presumed to be primarily due to the loss of NPs with the use of a 25 μm filter for the mock trial. FTIR analysis of the MPs obtained from both layers of the density separation was compared to the IR spectra of the individual commercial plastics. The IR spectra from the mock trial allowed observation of a spectrum produced from a mixture of the standard plastics and provided us with an idea of how the peaks of each standard plastic would influence each other. As presented in **Supplementary Figures 4C–E**, the spectra obtained for the mock trial displayed the dominating peaks for each of the four commercial plastics as expected. The peaks for PP at 2950 cm^-1^ and 1375 cm^-1^ were clearly visible, as well as the peak for PE at 2850 cm^-1^. The peaks for PET at 1715 cm^-1^ and 1095 cm^-1^ were easily distinguished from others. The peaks for PS were slightly weaker in intensity, which agreed with the scans taken of the commercial polymers. Nonetheless, the peaks for PS at 3025 cm^-1^ and 690 cm^-1^ were observed. This data confirmed that the individual plastics could be identified and distinguished from a mixture using the dominating FTIR peaks listed in **Table 1**. Precautions were taken to prevent extensive plastic decomposition and functionalization during the extraction procedure by controlling our reaction time, temperature, concentration, and pH carefully. A small signal at 3300 cm^-1^ in the IR spectrum may indicate some moisture is absorbed during extraction. The FTIR of the MPs obtained from the mock trial provides evidence that little to no degradation or functionalization occurs to the plastics during the extraction procedure.

Analysis of the IR spectra obtained for the top layer of the density separation indicated that the MPs were of PP, PE, PS, and PET origins (**Supplementary Figures 4C–E**). FTIR analysis of the bottom layer revealed that it was primarily composed of PET particles (**Supplementary Figures 4F**). This suggests that PET was the only MP that was lost in a significant amount during the extraction (7.8% of total plastics, 32% of total PET added to the mock trial). This is not surprising, as PET and the brine solution used for the density separation have similar densities. The MPs in the top layer were further analyzed by DANS staining and viewed under a UV LED and imaged using a laser scanning confocal microscope. UV illumination alone confirmed the FTIR analysis and demonstrated that the colors of the individual plastics remained discernable even in a mixture. Similar to what was observed in the commercial plastics, each plastic presented a unique color in the mixture after the staining process: PP: blue, PE: green, PS: yellow, PET: red (**Supplementary Figure 4G**). Consistent results were obtained when the DANS-labeled MPs were observed in higher definition under a fluorescence confocal microscope (**Supplementary Figures 4H–J**). In line with the findings using UV luminescence, the individual fluorescence spectra for the four plastics could be separated using fluorescence confocal microscopy allowing imaging and specific identification of the MPs of PP, PE, PS, and PET origins in liquids after extraction.

SEM and XRD were also performed on the MPs obtained from the mock trial to confirm the IR and imaging results. The SEM images of the MPs obtained for the mock trial revealed polymeric material similar to that seen in the SEM of the commercial polymers (**Figure 2I–J**). This suggests minimal degradation or functionalization occurred to the plastics during the extraction procedure. The XRD data of the mixture obtained from the mock trial revealed that all four of the commercial polymers could be identified. The signal intensity was diminished, which may be due to the small amount of sample and the sample being a mixture. Nonetheless, the plastics were able to be identified indicating minimal decomposition and functionalization during the extraction procedure (**Figure 2**). This is in agreement with what was seen in FTIR. Together, these data confirm the validity of our extraction and identification methods.

### Characterization of microplastics in water samples collected from the natural environment

**Figure 3:**
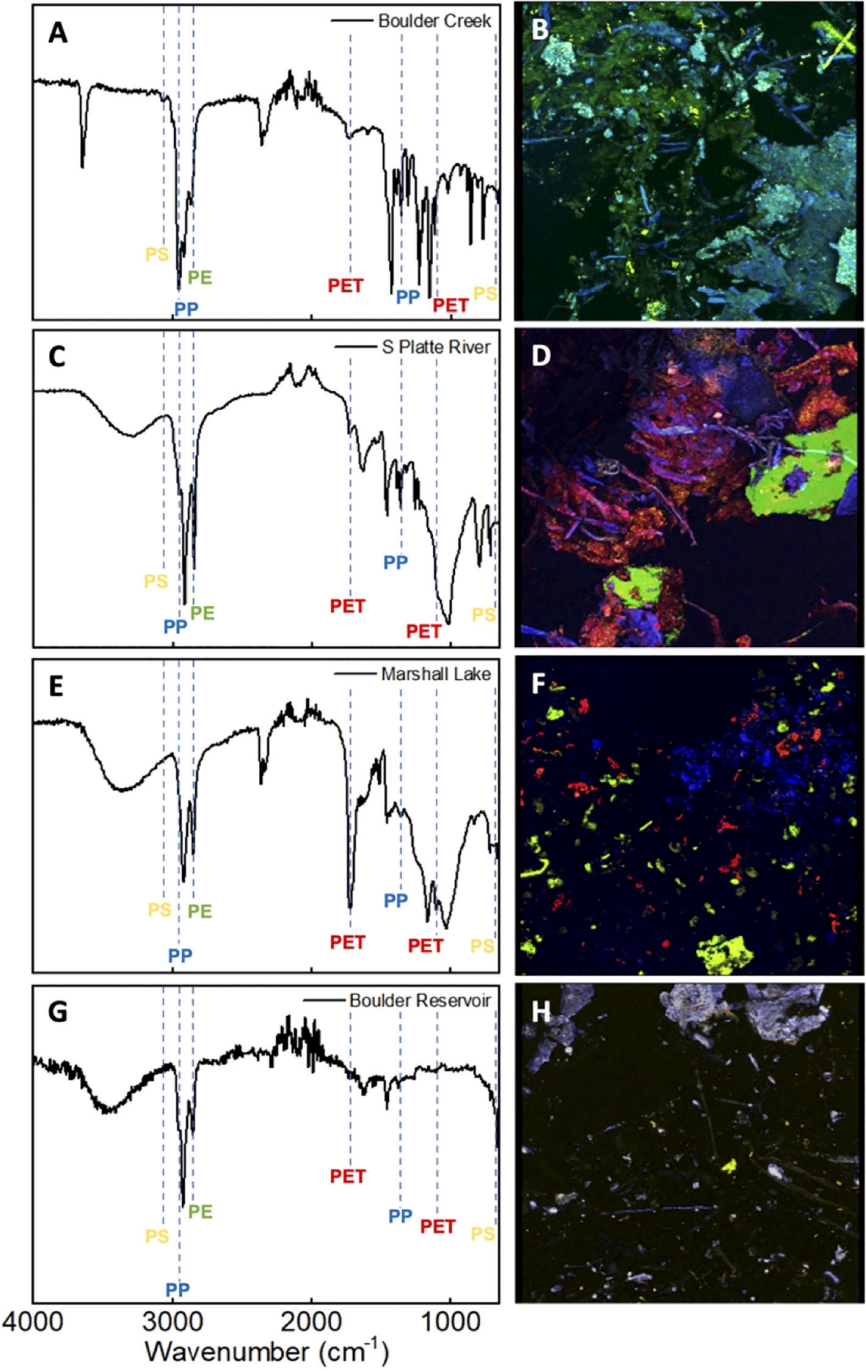
FTIR spectra and fluorescent confocal microscopy images of the environmental MPs obtained from local water sources surrounding the Greater Denver area. **(A, B)**: Corresponding FTIR spectrum **(A)** and image of stained environmental MPs **(B)** obtained from Boulder Creek. **(C, D)**: Corresponding FTIR spectrum **(C)** and image of stained environmental MPs **(D)** obtained from South Platte River (S Platte River). **(E, F)**: Corresponding FTIR spectrum **(E)** and image of stained environmental MPs **(F)** obtained from Marshall Lake. **(G, H)**: Corresponding FTIR spectrum **(G)** and image of stained environmental MPs **(H)** obtained from Boulder Reservoir.

Water samples were collected from multiple water sources in the Greater Denver Area, including Boulder Creek, Boulder Reservoir, Marshall Lake, and the South Platte River. Tap water from the University of Colorado campus and commercial bottled water samples were also collected. Next, the MPs were successfully isolated using the methods discussed above. As shown in **Supplementary Figure 5A–D**, the amount of MPs extracted varied among the water sources. The findings listed are in descending order of greatest to least volume: Marshall Lake had the most microplastics per volume, averaging approximately 1.47 mg/L, South Platte River had approximately 0.17 mg/L, Boulder Creek 0.12 mg/L, and Boulder Reservoir 0.059 mg/L. Tap and bottled water samples had the least microplastic volume with levels that were too insignificant to allow further analysis with FTIR, imaging, or biological study. FTIR analysis was performed on the extracted environmental MPs once dried. As expected, the IR spectra of the samples highly resembled the mock trial spectra and showed signals that matched the commercial polymers. The IR spectra of the environmental samples were analyzed for each MP using the selected identifying peaks as listed in **Table 1**. **Figure 3** and **Supplementary Figure 5E** depict the IR spectra. **Supplementary Figure 6** depicts the IR spectra obtained for each environmental location directly overlaid with the commercial polymer spectra for comparison. **Figures 3A, C, E, G** show that all samples exhibited peaks corresponding to PE and PP as indicated by peaks at 2850 cm^-1^ for PE and 2950 cm^-1^ and 1375 cm^-1^ for PP. Marshall Lake and South Platte River showed signals for PET with peaks at 1715 cm^-1^ and 1095 cm^-1^. Boulder Creek showed a peak at 1715 cm^-1^ indicating PET; however, the second standard peak was absent making it difficult to determine from IR alone if PET was present. Boulder Creek and Boulder Reservoir displayed signals for PS at 690 cm^-1^ and 3025 cm^-1^. As seen in the IR, a small amount of functionalization may have occurred to the environmental MPs shown by the differing peaks above 3000 cm^-1^, which may indicate an increase in hydrophilicity. However, this could be due to moisture being absorbed during the aqueous extraction. It has been previously reported that UV-weathered PET samples underwent oxidation and degradation (53). It has also been reported that all four commercial polymers can be degraded under environmental conditions (53–56). This suggests that environmental MPs exposed to sunlight and an aqueous environment containing bacteria and microbes undergo oxidation naturally. Due to the lack of evidence for functionalization in the MPs obtained from the mock trial, any functionalization observed is likely to have occurred in the environment.

As described above, DANS staining and fluorescence confocal microscopy were undertaken, allowing a complementary visual analysis and confirmation of the results from the FTIR analysis (**Figures 3B, D, F, H**). The fluorescence spectra were analyzed and compared to the standard for determination of polymer identity. Visual inspection allowed the amount of individual plastics in the environmental samples to be qualitatively quantified. The microscopy images of the Boulder Creek MPs exhibited large amounts of blue and green polymers representative of PP, PE, as well as a small amount of yellow polymers indicative of PS (**Figure 3B**). Although the IR of Boulder Creek also showed a minor peak around 1715 cm^-1^, it is missing the second standard PET peak in its IR spectrum. The confocal imaging did not observe any PET, providing strong evidence that the sample did not contain a significant amount (**Figure 3B**). The imaging of South Platte River (**Figure 3D**) indicated relatively even amounts of blue, green, and red polymers representative of PP, PE, and PET. Similarly, the images obtained for Marshall Lake indicated comparative amounts of blue, green, and red polymers representative of PE, PP, and PET (**Figure 3F**). Boulder Reservoir displayed a large number of plastic particles with fluorescence spectra representative PP shown by blue polymers. The sample also displayed a small amount of yellow and green polymers indicating the presence of PS and PE (**Figure 3H**). However, the amount of PE in the sample was relatively small compared to PP and PS, making it difficult to discern from the confocal images.

Further analysis of the confocal images revealed that the morphology and size distribution of the MPs from each water source differed. A large amount of aggregation and small polymer fusion was observed in some samples. Boulder Creek displayed large aggregates of PP and PE, as well as smaller sized particles of PS and PP (**Figure 3B**, **Supplementary Figure 7A, B**). South Platte River showed the largest amounts of aggregation indicated by larger particles and overlapping plastics shown by areas of blending colors (**Figure 3D**, **Supplementary Figure 7C, D**). Marshall Lake showed almost no aggregation indicated by smaller plastic particles and a more even particle size distribution (**Figure 3F**, **Supplementary Figure 7E, F**). Boulder Reservoir showed large aggregates of PP and more evenly sized particles of PE and PS. However, there was less overlap or aggregation between different plastics compared to Boulder Creek and South Platte River. This is likely due to the extremely small amount of polymeric material (**Figure 3H**, **Supplementary Figure 7G, H**). This aggregation or small polymer fusion may be due to the heat used during the staining process or during the drying process after staining as outlined in the procedure and previous literature (41). Of interest, this phenomenon appears to be less significant in the Marshall Lake sample (**Figure 3F**). This may be due to the large volume of plastics isolated in that sample. This variation does not hinder our ability to identify each plastic in the environmental samples, but does limit the ability to quantify the amount of each plastic accurately and precisely.

SEM and XRD were also performed on the environmental plastics. The SEM images of the environmental polymers show some differences when compared to the commercial polymers, however the individual polymeric structures are identifiable. The SEM images show that our polymer samples are within the desired size limits and there is a range of microplastics present in the samples. For polymers with long strands like PE and PP, strands longer than 300 μm were able to pass through the pores in rare instances (**Supplementary Figure 8**). Some surface degradation may be visible in the environmental polymers, however based on the results of the mock trial it likely occurred in the environment. Furthermore, the images of the environmental samples obtained resembled the imaging done through confocal microscopy. Larger pieces were obtained for South Platte River and Boulder Creek. Marshall Lake consisted of mostly smaller particles. Lastly, Boulder Reservoir had limited material but showed larger and smaller pieces that resembled PP and PS, in agreement with the microscopy study. XRD analysis on the environmental samples did not provide any significant data. This may be due to the extremely small amount of material available for testing or that the samples were subjected to extreme conditions in the environment.

### Microplastics from environmental water samples change cell morphology and induce inflammatory responses in human cells

**Figure 4:**
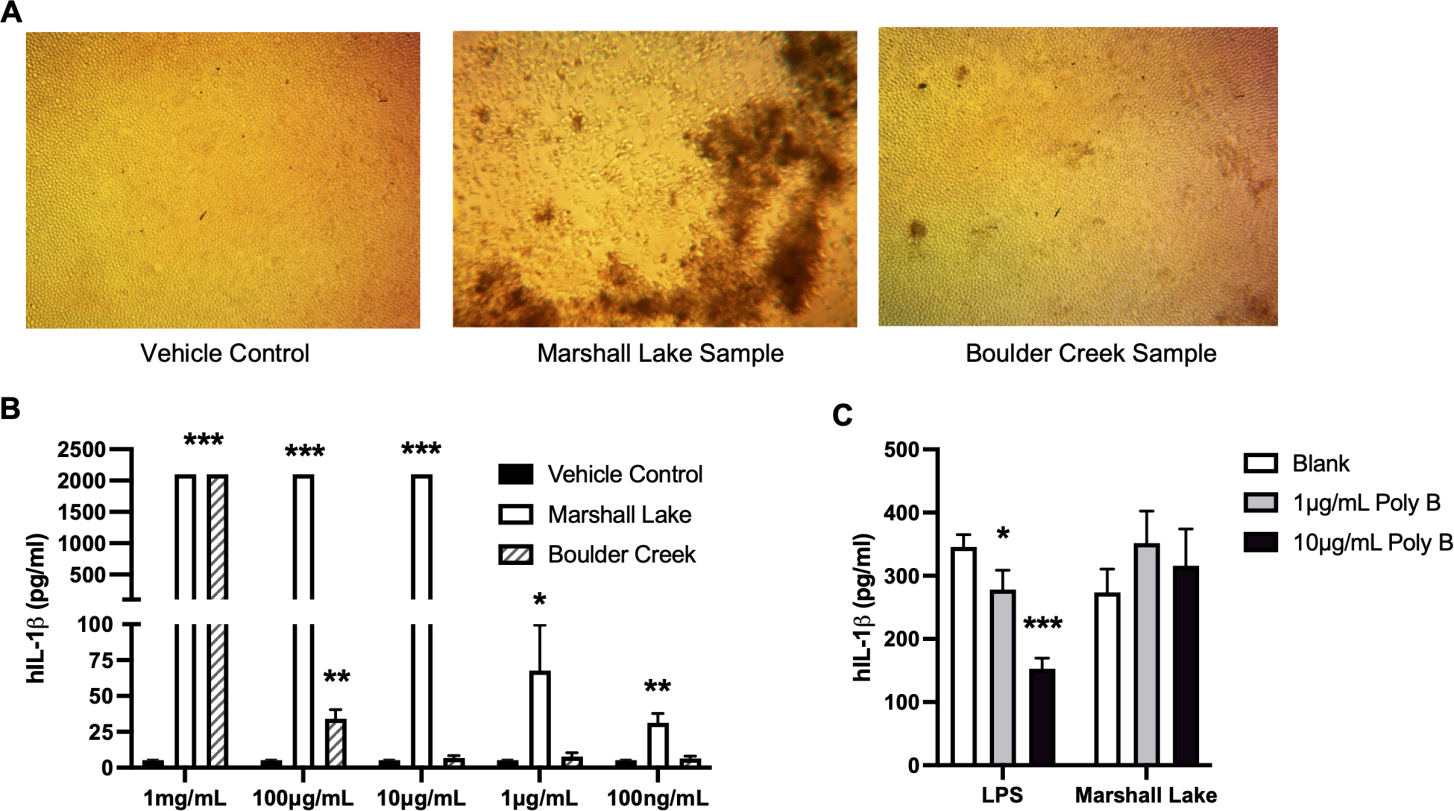
MPs from natural environment induce human cell inflammation. **(A)** Effects of the MPs on human cell morphology. **(B)** Dose responses of the MPs from the natural water samples on human cell inflammation. **(C)** Endotoxin inhibitor Polymyxin B (Poly B) does not block the inflammatory effects of the MNPLs on human THP-1 cells. Human monocytic THP-1 cells were treated for three to five days with MPs from the natural water samples. N = 3, Mean ± SD, *P < 0.05, **P < 0.01, ***P < 0.001 compared to vehicle control.

Various toxicological effects of MNPLs have been reported in different animal species (17–20). However, it is unknown how such particles directly impact human health and human cell physiology. Like other foreign substances, MNPLs, when entering the human body, are first encountered by the innate immune system in which inflammation is our body’s first line of defense (57–59). We therefore first assessed the inflammatory effects of the original microplastics collected from the environmental water samples on human cells. MPs collected from Marshall Lake and Boulder Creek were selected based on the type, quantity, and size distribution of the plastic particles under a microscope, compared to MPs collected from South Platte River and Boulder Reservoir. Furthermore, this enabled a comparative analysis of MPs from a commercial water source and a recreational water source which revealed differences in the major plastics detected. As shown in **Figure 4A**, MPs collected from Marshall Lake significantly altered the cell morphology and reduced cell density of human monocytic THP-1 cells. No obvious effect on cell morphology was observed for MPs collected from Boulder Creek. Interestingly, the microplastics collected from Boulder Creek and Marshall Lake both robustly induced inflammatory IL-1β and IL-6 secretion in the cells and the inflammatory responses increased with prolonged incubation time (**Supplementary Figure 9**). In a dose-response study with concentrations tested between 100 ng/mL to 1 mg/mL (**Figure 4B**), the microplastics collected from Marshall Lake showed a strong dose-dependent response and were effective in inducing IL-1β at a dose as low as 100 ng/mL. The microplastics collected from Boulder Creek presented a strong inflammatory effect at 1 mg/mL which gradually reduced at 100 μg/mL and disappeared at 10 μg/mL. The endotoxin inhibitor Polymyxin B (60), did not block the inflammatory effects of the microplastics on human THP-1 cells (**Figure 4C**), excluding the possibility of potential endotoxin contamination during microplastic purification.

### MNPLs from commercial sources induce inflammatory responses in various human cells and tissue

**Figure 5:**
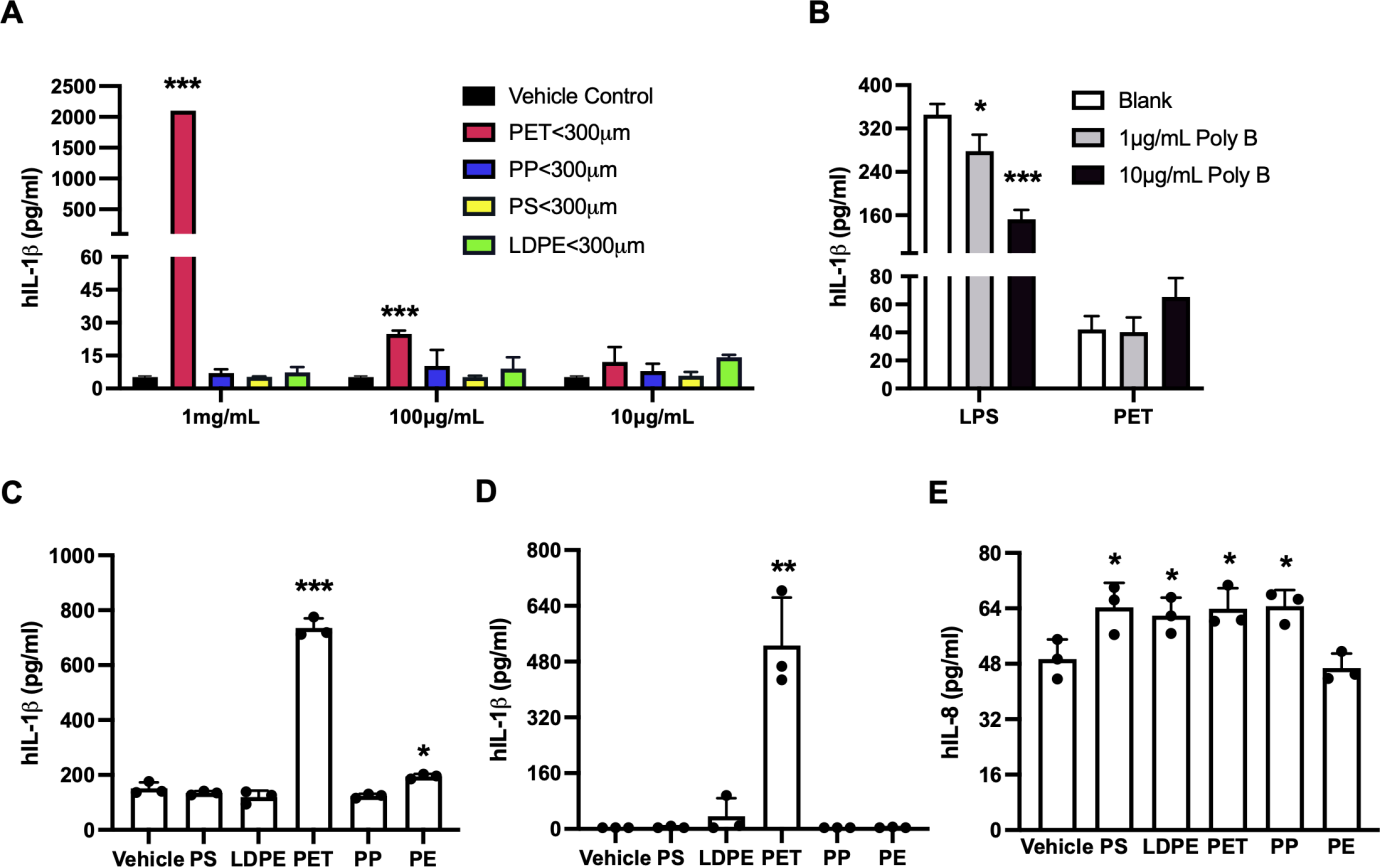
MNPLs especially PET from commercial sources induce human cell inflammation. **(A)** MNPLs induce inflammatory IL-1β production in human monocytic THP-1 cells. **(B)** Endotoxin inhibitor Polymyxin B does not block the inflammatory effects of MNPLs on human THP-1 cells. **(C)** MNPLs induce inflammatory IL-1β production in human PBMC culture. **(D)** MNPLs induce inflammatory IL-1β production in human whole blood culture. **(E)** MNPLs induce inflammatory IL-8 production in human T84 intestinal epithelial cells. The cells or whole blood cultures were treated for three to five days with or without MNPLs from commercial sources. N ≥ 3, Mean ± SD, *P < 0.05, **P < 0.01, ***P < 0.001 compared to vehicle control.

To identify the specific type (s) of MNPLs that mediate the inflammatory function in the water samples, we next characterized the effects of the commercial MNPLs of PP, PE, PS, or PET origin on inflammation in human THP-1 cells. As shown in **Figure 5A**, the commercial MNPLs of PET origin robustly induced inflammatory IL-1β release in the THP-1 cells and demonstrated a strong dose-response similarly to the MPs from environmental water samples. The inflammatory response remained intact in the presence of endotoxin inhibitor Polymyxin B (**Figure 5B**). However, unlike MPs collected from Marshall Lake which significantly impacted cell morphology and reduced cell density (**Figure 4A**, **Supplementary Figure 10A**), the MNPLs of PET origin did not alter cell morphology visibly (**Supplementary Figure 10A**). Considering that recent studies reported significant amounts of MNPL particles in the blood and feces of healthy people (6, 7), we next conducted experiments to explore the direct impact of the MNPLs on inflammation in human blood immune cells, whole blood, and human intestinal epithelial cells. The human peripheral blood mononuclear cells (PBMCs) and whole blood cultures are rich in various primary immune cells including monocytes and lymphocytes. We examined the inflammatory effects of the commercial MNPLs in PBMCs and whole blood cultures from healthy donors, two assays we routinely use to study innate immune responses (61, 62). Similar to what was observed in the monocytic THP-1 cells, commercial MNPLs with PET origin triggered a robust inflammatory response in the primary PBMC and whole blood cultures (**Figures 5C, D**). Again, unlike the MPs collected from Marshall Lake, commercial MNPLs with PET origin had no significant effect on the cell morphology of PBMCs (**Supplementary Figure 10B**). Next, we assessed the inflammatory impacts of MNPLs in human intestinal epithelial T84 cells. As shown in **Figure 5E**, we detected increased secretion of the inflammatory chemokine IL-8 in response to commercial MNPLs. Levels of IL-1β and IL-6 were undetectable in T84 cells (data not shown), which is consistent with an earlier report (63).

### MNPLs of commercial and environmental sources induce human cell apoptosis and necrosis

**Figure 6:**
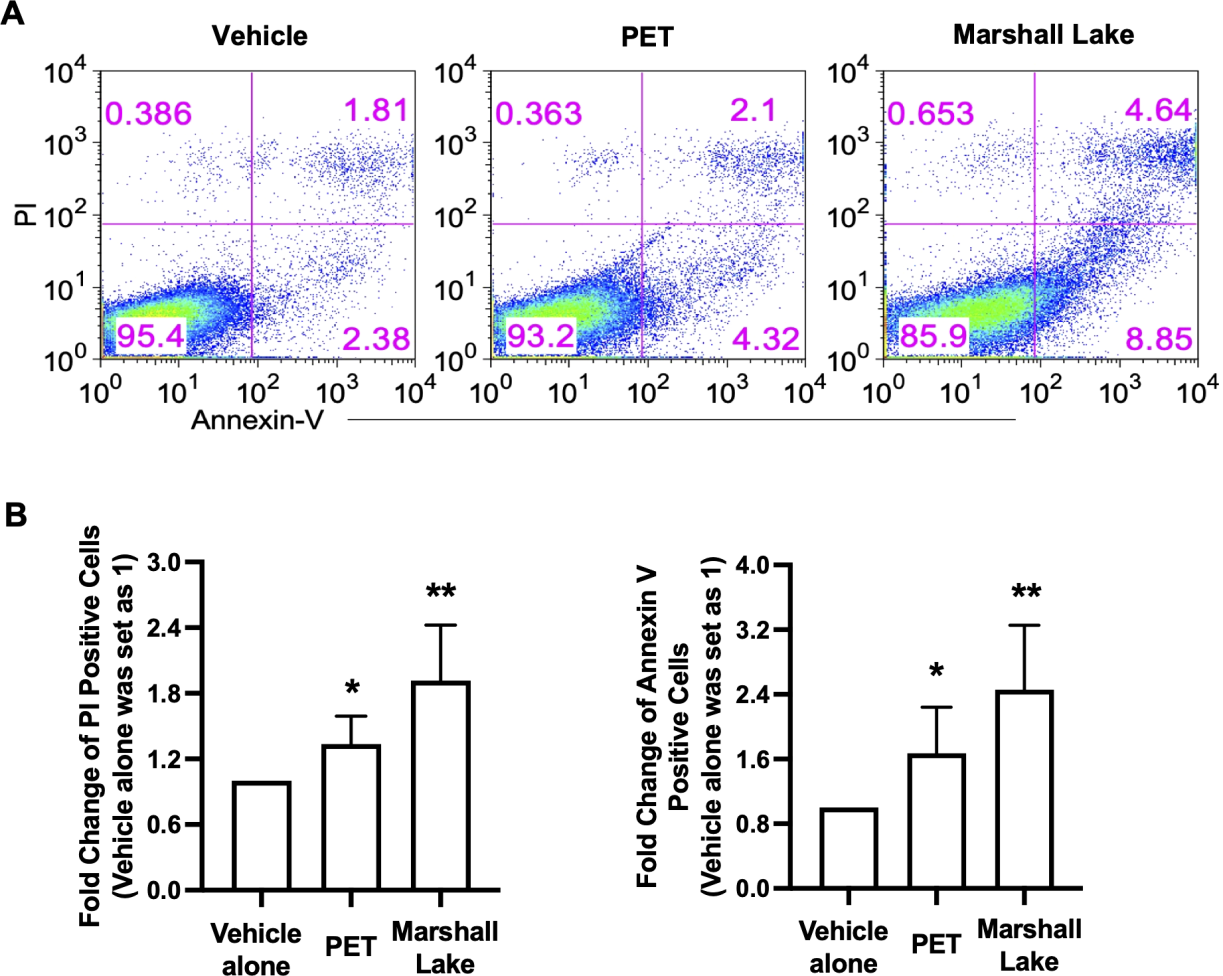
MNPLs induce human cell death. **(A)** Effects of commercial MNPLs from commercial sources or MPs from environmental water resources on human cell death. Human THP-1 cells were treated with or without MNPLs from commercial (PET synthetic) or MPs from environmental water resources (Marshall Lake) for five days before the cells were stained with PI and Annexin V for flow cytometry analysis. **(B)** Statistical analysis of the flow cytometry data. Fold change of PI or Annexin V positive cells in vehicle alone- or PET- or plastics from Marshall Lake-treated samples over vehicle alone- treated samples (vehicle alone- treated samples were set as 1). N = 3, Mean ± SD, *P < 0.05, **P < 0.01 compared to vehicle control.

Given that MPs collected from Marshall Lake significantly changed the cell morphology of human THP-1 cells and PBMCs (**Figure 4A**, **Supplementary Figure 10**), we next carried out flow cytometry experiments to characterize the influence of the commercial and environmental MNPLs on cell death. The effects of commercial MNPLs of PET origin and environmental MPs from Marshall Lake were compared in parallel in human THP-1 cells. The cells treated with or without the plastic particles were stained with fluorophore-conjugated Annexin V and PI to differentiate the stages of cell death: cell apoptosis (Annexin V positive) and necrosis (PI positive). As shown in **Figure 6**, both commercial MNPLs of PET origin and MPs collected from Marshall Lake enhanced cell apoptosis (Annexin V positive staining) and necrosis (PI positive staining), in comparison to vehicle control-treated cells. Not surprisingly, MPs collected from Marshall Lake induced more cell death than the commercial MNPLs of PET origin, which is in line with the results from the cell morphology and inflammation study. The MNPLs of PET origin also induced cell apoptosis in human T84 cells as observed under a fluorescent microscope where the cells were stained with FITC-conjugated Annexin V (**Supplementary Figure 11**). However, consistent with the observations in THP-1 cells, the PET plastics did not affect the cell morphology visibly.

## Discussion

Maintaining high water quality is fundamental for the health of the ecosystem and for preserving life on our planet. In a technologically advanced society this has become increasingly difficult due to our reliance on manufactured products such as plastics. Only recently has the ubiquitous presence of plastics and their degraded products, MNPLs, been recognized as an urgent planetary threat. As plastic production continues to escalate and a new era of nanotechnology beckons, there is an urgent need to characterize the composition and health risks of MNPLs in our environment.

Given their small size, separation and extraction of MNPLs can be challenging making obtaining a biologically relevant sample concentration problematic (64–66). As such, the objectives of this study were achieved in three steps. First, we generated new methods to extract and identify individual MPs from a mixed aqueous sample. Second, we extracted MPs from environmental water sources and characterized their plastic composition using both chemical and imaging analyses. Third, we assessed the direct effects of the environmental MPs in comparison to the commercial MNPLs on human cell inflammation, cell morphology, and cell survival.

In pursuit of our first objective, synthetically pure MNPLs of PP, PS, PE and PET were produced from commercial sources and analyzed with FTIR and confocal imaging to establish the standards for evaluation of the mixed plastic samples. The commercial samples were then analyzed with DLS, SEM, and XRD for structural analysis and confirmation of size. DLS data revealed coagulation occurs in the commercial MNPL solutions over time. Nonetheless the data confirmed the particles in solution remained below the target size limit. It should be noted that our intent was to mimic environmental conditions, and while coagulation was observed in our artificial environment, it likely would be seen in natural environments as well. The SEM performed on the commercial polymers confirmed the MNPLs were within the expected size ranges and their polymeric structures were intact. The XRD data matched previously reported data in literature and confirmed that the commercial polymers used for the cell testing were extended polymeric structures.

To develop a standardized procedure for extracting and identifying individual plastics in a mixed aqueous sample, a mock trial was used. The luminescence as well as the imaging results from the mock trial correlated nicely with the FTIR analysis of the plastic composition (**Supplementary Figures 4G–J**). Our findings indicate that FTIR analysis can reliably determine the presence of polymers but not their amounts, whereas UV illumination alone can broadly identify plastic composition in the stained samples, and fluorescence spectra can specifically identify individual plastics allowing for qualitative quantification. Spectral phasor analysis for quantitative analysis was performed on the fluorescence images obtained from the mock trial and four separate signals could be seen in the phasor plot. However, the ratio of the polymers changed significantly depending on the aliquot of MNPLs used. Due to this non-uniform mixing it was determined that the polymer ratios calculated were unreliable. As described in the Results section, we found a significant amount of plastic material was lost during the extraction process: 7.8% of PET due to its density and 7.2% of smaller MPs (< 25 μm) and NPs due to filtration. Therefore, the amount of MPs extracted from environmental water samples would not be representative of the total MNPL in the samples. It would, however, give us a rough estimate and allow for comparison of the amounts between sources. The SEM of the mock trial confirmed that the extraction procedure for the environmental polymers was successful. The XRD patterns of each commercial polymer were distinguishable in the XRD pattern obtained for the mixture of MPs from the mock trial. This confirmed that the crystallinity of these polymers was maintained after the extraction procedure, although the intensity of the peaks was reduced. This may be due to the sample being a mixture and the quantity of each polymer being reduced compared to a pure sample. At the present, it remains difficult to obtain 100% of the MNPL particles, specifically NPs, from environmental water sources due to filtration limitations and density concerns. Future studies with more rigorous separation techniques are needed for a more accurate quantification of the MNPLs in environmental samples. This increased accuracy will enable us to determine biologically relevant concentrations for future studies with MNPLs and their health risks.

In pursuit of our second objective, we selected four different water sources for analysis, as they represented major commercial and recreational water sources in the area. As stated above, these locations were chosen for accessibility and differences in major function. As before, FTIR was the initial probe into determining the identity of the MPs contained in the samples. Further analysis with DANS staining and fluorescence confocal microscopy was undertaken, allowing a complementary visual analysis. The SEM of the environmental samples showed polymeric material similar to what was seen in the mock trial (**Supplementary Figure 8**). Individual polymeric structures could be identified and the plastics were within the size limits of the sieves used. In some cases, long strands of either PE or PP made it through the pores of the sieves, however, these were rare. The XRD of the environmental samples did not provide any useful data, which may be due to the small amount of material or some degradation of the polymers. However, as mentioned above, the lack of degradation observed in the MNPLs obtained from the mock trial, suggests any degradation observed in the environmental samples likely occurred in the environment before extraction.

Analyzing the data allows for some interesting speculations as to why some water sources contain more of certain types of plastics. All four water sources showed signals for PE and PP. This is not surprising as these plastics are widely used, with PE being the most common plastic on earth (67). PET, a plastic used in manufacturing, packaging, and containers was isolated in two of the water sources: Marshall Lake and South Platte River. Both water sources are commercially used with South Platte River having industrial and agricultural runoff. Marshall Lake had the most MPs per volume. This could be explained by the Marshall Fire of 2021. Fire water and combustion product runoff flowed into the lake. PS, a plastic used for food transport and storage was isolated in two of the water sources: Boulder Creek and Boulder Reservoir, both recreational water sources. This is not surprising as Boulder Creek serves as a runoff for the cities of Boulder and Denver. Boulder Reservoir is a source of drinking water for Boulder City and, as expected, had the lowest concentration of MPs per volume. The absence or extremely small amount of PET in the recreational water sources could indicate this plastic is isolated to commercial sources. These basic observations may enable the categorization of water source pollution and facilitate targeted interventions to mitigate plastic pollution and waste.

In pursuit of our third objective, the direct influence of the environmental and commercial MNPLs on human cell inflammation, cell morphology and cell survival were assessed in various human cells including human cell lines and primary human PBMCs, as well as whole blood cultures. Marshall Lake representing a commercial water source and Boulder Creek representing a recreational water source were selected for side-to-side comparison due to their significant difference in plastic composition. Interestingly, we observed varied inflammatory responses between the two environmental water sources. We found that the MPs of Marshall Lake containing large amounts of PET were significantly more powerful in triggering inflammation in the cells than MPs of Boulder Creek containing mostly PP. Similarly, cell morphology was affected by the MPs from Marshall Lake, but not by MPs from Boulder Creek. And MPs collected from Marshall Lake induced a stronger dose response in inflammation than MPs collected from Boulder Creek. Altogether, these data confirmed a correlation of the composition of the plastic particles in environmental water sources with their biological activity. This finding is in line with what we observed in the commercial pure plastic samples where MNPLs of PET origin presented the strongest inflammatory effect among the four types of plastics tested. The exact mechanism remains unclear. However, it may be related to the unique chemical structure of the polymers and their recognitions by the innate immune system. In addition, although we observed that environmental MNPLs rich in PET can alter human cell morphology under a phase-contrast microscope, further studies are needed to fully characterize the impacts of MNPLs on cell morphology and differentiation. In summary, the comparison of inflammatory responses from the Fenton-treated environmental plastics to the pure commercial plastics showed similar inflammatory responses based on plastic composition. Specifically, environmental samples containing mostly PP, PE, and PS did not show extensive inflammatory responses, whereas the commercial PET samples and the environmental samples rich in PET both showed robust inflammatory responses. This provides evidence that any amount of functionalization that occurred in the environment, did not have a significant effect on the results we observed. Although, it may explain the slight increase in inflammatory response observed with environmental plastics, which may contribute to the environmental concern of MNPLs.

It is noteworthy that both MPs from Marshall Lake rich in PET and the commercial MNPLs of PET origin can induce cell death. The MPs from Marshall Lake changed human cell morphology significantly as observed under microscope. However, PET alone did not change cell morphology visibly. This indicates that PET is not the only biologically active substance in the MPs from Marshall Lake. Moreover, the mixed MPs from Marshall Lake induced more cell death than the commercial MNPLs of pure PET origin. Together, our results indicate MPs from environmental water samples in general pose stronger detrimental effects on human cells than pure commercial MNPLs. This may be due to their mixed plastic origin or possible contaminants on the surface. In fact, microplastics from environmental water samples are of mixed origins and may act synergistically. Moreover, many plastics incorporate chemical additives such as Bisphenol A (BPA), flame retardants and antimicrobial agents increasing potential toxicity (32). Although examination with Polymyxin B excludes the possibility of endotoxins or bacteria, these small plastic particles can become biofilms, adsorbing surfaces, sinks or carriers for volatile organic compounds (VOCs), heavy metals, and other contaminants in the environment potentially exacerbating inflammation (28–33). Release of the VOCs and heavy metals from polymeric materials occurs upon heating (33). This indicates some of the VOCs and heavy metals adsorbed onto the surface of the environmental polymers may have been released during the oxidation of the material. Further, while the oxidation treatment and density separation during the extraction process removed any freely dissolved organic and inorganic contaminants from the environmental samples, it cannot be ruled out that some environmental pollutants and chemical additives that are adsorbed onto the polymer surface or integrated into the polymer structure may remain after extraction. Furthermore, when spectral phasor analysis was performed on the environmental samples, the phasor plot displayed four signals, one for each polymer, as before, however the cloud of points for each polymer were larger indicating the spectral width had changed. This may indicate the surface of the environmental plastics was different than the commercial samples. Current research has shown that mixtures of MNPLs and VOCs are more toxic to living cells than the pure compounds, however the results are inconsistent (32). For example, Fu et al. found that copper with PVC MPs had significant increases in several toxicity parameters after ten days (68). On the other hand, Davarpanah et al. found that the toxicity of copper did not increase in the presence of PE MPs (69). This illustrates that the identity, size, and concentration of the MNPLs being studied, as well as exposure time are important variables when gathering significant data. Additional investigation into the development of methods for separating plastics from other non-plastic small particles is needed to avoid having a mixture of contaminants in the environmental samples and to isolate the specific effects of environmental MNPLs on inflammation or disease. Similarly, further research into defining methods where specific plastic contaminants can be separated, identified, and assessed to distinguish their effects from that of the MNPLs themselves would be of great interest.

In human intestinal epithelial T84 cells, MNPLs of all origins elicited similar inflammatory responses (IL-8). Unlike the THP-1 cells and PBMCs where MNPLs of PET origin induced robust amount of IL-1β, IL-1β production in the T84 cells were below detection. The different inflammatory responses to MNPLs between the macrophages and intestinal epithelial cells may be due to their distinct cell recognition mechanisms for plastic particles or intracellular inflammatory pathways. It is noteworthy that our inflammatory and cell apoptosis data of the intestinal cells align with several recent findings in which various plastic particles can affect the distribution of gut microbiota and inflammation, perturb colonic epithelial homeostasis and induce intestinal barrier dysfunction (19, 70, 71). Notably, fecal microplastics were found to be substantially more abundant in patients suffering from IBD than in healthy individuals (7). Altogether, these findings indicate a positive correlation between MNPLs exposure and the development of intestinal diseases. However, the underlying mechanism remains unknown and is worthy of further exploration.

**Figure 7:**
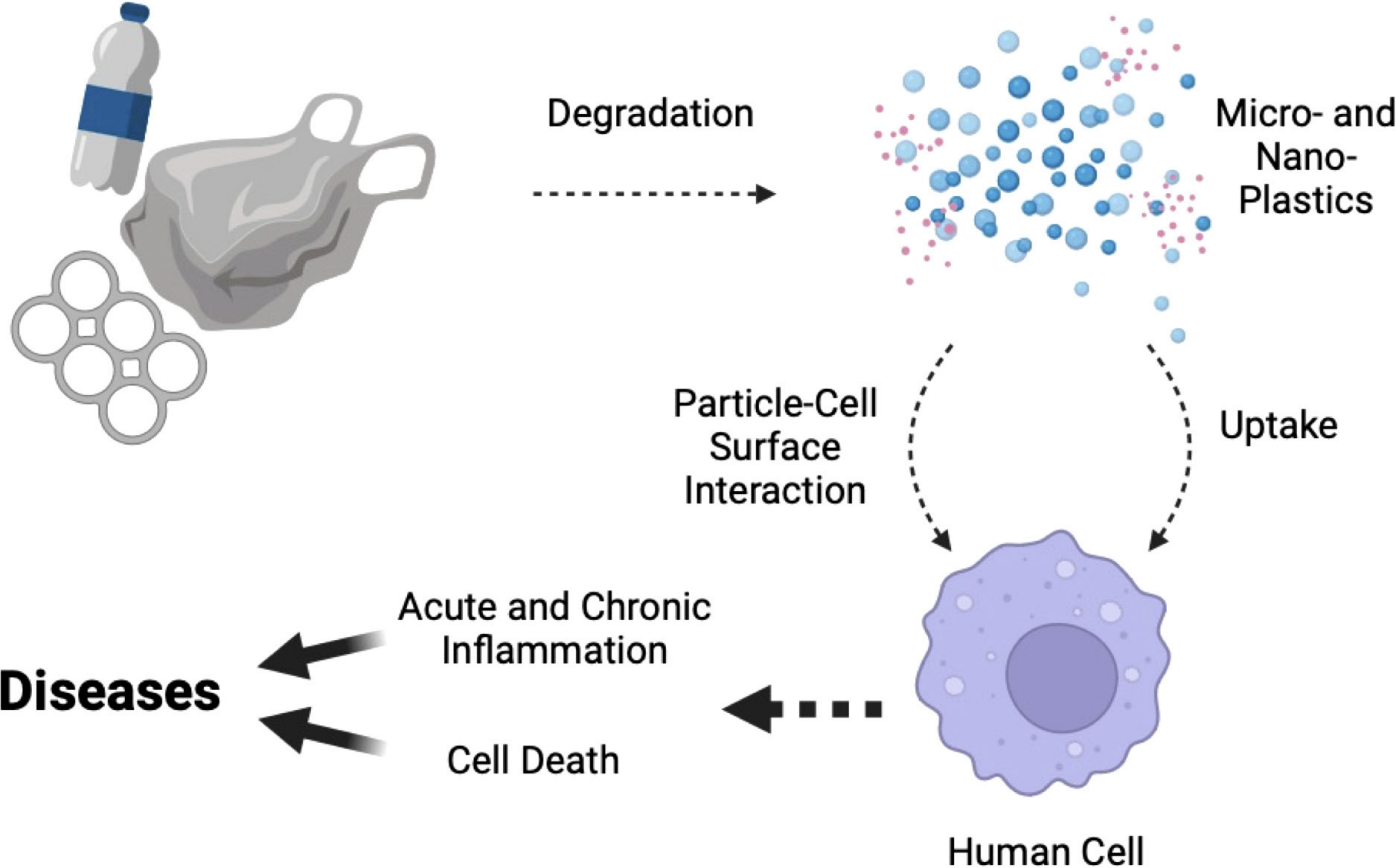
A schematic diagram describing the impacts of chronic MNPL exposure on human cell inflammation and cell death, and their correlation to potential disease development. Debris from human-consumed plastics undergoes degradation over time, breaking down into MNPLs. MNPLs, when exposed to human cells, may directly stimulate the cells through interactions between the particles and the cell surface, or the cells can uptake small enough particles, triggering intracellular signaling pathways such as inflammation or cell death. Chronic exposure of the cells to MNPLs can lead to chronic inflammation and cell death, which, in the long run, may contribute to disease development. The diagram was created with BioRender contents.

In summary, in the present study, we combined chemical identification and biological assessment to first extract and identify environmental MPs from ecological water sources, and then compare their inflammatory effects in human cells in parallel to pure MNPLs from commercial sources. The doses used in the cell cultures may be higher than the daily amount we intake from food or drinks; however, MNPLs could potentially accumulate in the human body (6, 7, 21–27) and can exert a chronic and additive effect on human health (**Figure 7**). In fact, it is important to note that even with such a short incubation time in our study, induction of inflammation and cell death was observed for commercial and environmental MNPLs rich in PET. Moreover, inflammation induced by the plastics increased in a time-dependent manner in the cell cultures, suggesting a potential concern for chronic inflammation due to accumulated MNPLs in the human body. This is consistent with earlier findings that MNPLs were observed in living human tissue/organs whereas significant amounts were detected in patients with chronic diseases including IBD, liver cirrhosis and cardiovascular diseases (7, 25, 27). Whether long-term exposure to MNPLs directly contributes to the development of these chronic diseases warrants further exploration. In addition, studies on how the cells interact with MNPLs of different origins, the cellular uptake of MNPLs and the subsequent downstream signaling, as well as how the MNPLs interact with the human body are desired and worthy of future investigation. It is noteworthy that this study focuses on environmental MPs as environmental NPs were unattainable due to filtration limitations. Nevertheless, recent literature has shown that NPs may be more abundant and hazardous to human health due to the ease at which they can be absorbed by cells (72). Future studies focused on the isolation or identification of NPs for specific cell testing are also of interest. Overall, the health effects of MNPLs are a growing concern, and further research is needed to fully characterize them and assess their impact on the environment, human health, and disease development.

## Materials and methods

### Antibodies and reagents

For cytokine measurements, the corresponding ELISA DuoSets kits for human IL-1β, TNFα, IL-6, and IL-8 were purchased from Bio-Techne (Minneapolis, MN). FITC-conjugated Annexin V antibody was from BioLegend (San Diego, CA). The Propidium Iodide (PI) solution was from Tonbo Biosciences (San Diego, CA). The Polymyxin B sulfate salt powder was purchased from Sigma Aldrich (St. Louis, MO).

### Human cell lines

Human intestinal epithelial T84 cells and monocytic THP-1 cells were obtained from ATCC (Manassas, VA). The T84 cells were grown in DMEM/F-12 medium (1:1) supplemented with 10% FBS and 1% Penicillin-Streptomycin, and 1% glutamax. The THP-1 cells were grown in RPMI medium supplemented with 10% FBS and 1% Penicillin-Streptomycin. The cells were maintained at 37°C cell culture incubator with 5% CO2.

### Preparation of standard MNPL particles from commercial sources

PP, PE, and PS were purchased in bulk from Sigma Aldrich. The standard PET was obtained from a clear commercial water bottle made out of the plastic (recycling code 01). These standard samples of pure PP, PE, PS, and PET origin were sized by making each a powder in an industrial grinder. The micro- and nano-sized powder samples obtained for each plastic were then dispersed in water and passed through a specific molecular sieve (300 μm, 100 μm, 25 μm, 0.4 μm) depending on the maximum size desired. The plastic that remained on the sieve was discarded and the water was evaporated under vacuum to afford the dry MNPLs with the size desired. The dried plastic samples were then stored in a 20 mL glass vial. This provided standard MNPL samples that were in the nano- and micro-size range. A small amount of each type of MNPL (40 - 50 mg) was kept for FTIR analysis, for use in the standardized mock trial to confirm the extraction method was viable for the environmental sources, and for staining and fluorescent imaging. The remaining dried MNPLs were then dispersed in a phosphate buffered saline solution, refiltered, and used for cell testing. Initial concentrations ranged from 0.2 - 10 mg/mL.

### Mock trial for extraction of microplastics from water sources

5 - 10 mg of each standard plastic (equal amount from each size range) were combined and dispersed into 3.5 Liter (L) of H_2_O. The water was allowed to sit for three days and shaken vigorously throughout that time to ensure the plastics were as evenly dispersed in the water as possible. The water samples were then filtered through multiple metal sieves of various sizes (300 μm, 100 μm, 25 μm) to obtain all the material between 300 - 25 μm. The extraction of microplastics was performed using an innovative combination of established methods (50, 51, 73). The Fenton Oxidation was performed on the solid material obtained in order to remove all the bio-organic matter (plant material, bacteria, micro-organisms). This was done using a source of iron (II), in this case iron (II) sulfate (20 mL of 0.05 M solution), and 20 mL of 30% hydrogen peroxide solution. Fenton oxidation was chosen as it has been reported to be milder than pure hydrogen peroxide oxidation and less damaging to polymer material than reported enzymatic, acidic, and basic digestion procedures (50). In order to prevent extensive plastic decomposition and functionalization the reaction time, temperature, concentration, and pH were controlled carefully. It has been previously reported that time scales of under four hours at 140°C, lead to little or no mass loss in the four polymers used in this study (51). For this reaction, the temperature was kept at or below 75°C and the reaction time was one hour or less, as outlined in the cited literature (73). Under these conditions it has been reported that the mass loss of plastics is under 5-10% (50–52). Our mock trial was in agreement with this showing a mass loss of 7.2%. Additionally, the concentrations of iron and H_2_O_2_ were controlled at levels that were previously reported to be non-damaging to the plastics (50, 51, 73). Lastly, previous research has shown, the optimal pH for the Fenton reaction to limit polymer degradation and functionalization is 3-4 (51, 74). For this reaction the pH was maintained at approximately 3.5 for the duration of the reaction. The pH was maintained using small amounts of H_2_SO_4_ and NaOH, as it has been previously reported that H_2_SO_4_ is less damaging to the plastics than mineral acids (50, 51). This was followed by density separation using a saturated NaCl solution, brine, to separate heavier particles, including inorganic salts and sediment particles, from the MPs that have low density and float in the brine solution. Although brine and PET have similar densities, NaCl was used for the density separation as it has been previously reported to be the most affordable, environmentally safe, and significantly dense to separate a majority of commercial plastics, compared to solutions of ZnCl_2_, NaI, and CaCl_2_ (75). Furthermore, we attempted a mock trial with a saturated ZnCl_2_ solution and the amount of plastic did not significantly increase and a small amount of PET was still lost. The sample was passed through the 25 μm molecular sieves one more time to collect all the MPs greater than 25 µm. MNPLs of sizes < 25 µm were not collected due to the large amount of water and the inability to separate them from the salt dissolved in the water. The plastics were allowed to dry on the molecular sieve and then collected using a forceps and spatula into a pre-weighed vial for characterization and use in cellular studies.

### Collection of microplastic particles from local water sources

Once the extraction method as described above was established using the mock trial, it was applied to purify microplastics from water samples in the natural environment within the Greater Denver area. In brief, three samples (9 gallons each) from each site (Boulder Creek, Boulder Reservoir, Marshall Lake, South Platte River) were collected using PP containers. The environmental water samples were then subjected to the separation procedure explained above to collect microplastics of sizes between 25 - 300 μm. The separation procedures were outlined in detail in **Supplementary Figure 3**.

### Fourier transform infrared spectroscopy

IR spectra were obtained on a Cary 630 FTIR spectrometer by Agilent Technologies in attenuated total reflection (ATR) mode, equipped with a diamond measurement interface and controlled by Microlab software. Spectra were acquired in the range 4,000-400 cm^-1^ with a resolution of 2 cm^-1^. Each measurement was the result of the average of 8 scans. The ATR diamond crystal was cleaned with 70% ethanol/water and a background measurement was performed between each sample. The spectra were obtained by placing a small amount of dry sample over the crystal, compressing the sample against the diamond to ensure good contact between the sample and the ATR crystal, and then taking the scan. An IR spectrum for each of the standard plastics was obtained for comparison to the environmental mixtures. IR spectra of each environmental sample were taken in order to give an initial estimate of how much and which types of plastics were present. In the case where there was a larger amount of plastics obtained, multiple scans may have been necessary. The IR spectra of the environmental samples was analyzed using the spectra obtained from the standard microplastic samples.

### Staining of MNPLs using 4-dimethylamino-4’-nitrostilbene

The staining procedure and the fluorophore used were referenced from a paper by the Vetri lab at the University of Palermo (41). The standard MNPL samples and the environmental MPs were dyed using a 500 µM stock solution of DANS in ethanol. 50 μL of the stock solution were added to a 25 mg/mL suspension of microplastics in deionized water, to obtain a final DANS concentration of 25 μM. Samples were incubated for one hour at 60°C under sonication. The solution was then evaporated on a hot plate at 80°C overnight to obtain the dry stained samples of MNPLs. RGB pictures of stained standard plastics, under UV excitation using a UV-LED flashlight, were simply recorded by an iPhone XR camera.

### Fluorescent confocal microscopy

Confocal images were acquired with an Olympus FV3000, high-end laser scanning confocal microscope using a 10 × 0.3 NA objective. Aliquots of DANS-stained standard MNPLs or environmental MPs dispersed in water were deposited on a glass bottom dish with a 20 mm bottom well using a disposable pipette. Measurement was acquired using laser excitation at 405 nm. Emitted fluorescence was acquired in photon-counting mode. Spectral detection has been performed using a bandwidth of 5 nm and a step size of 3 nm in the range 450 – 650 nm. The scan area ranged from 1000 x 1000 to 256 × 256 pixels and the scan speed was 12 μs per pixel. Scans of each standard, as well as the mock trial were taken in order to check the validity of the method before dying and scanning the environmental samples. 3D confocal images were obtained for the mock trial and the environmental samples. Images were analyzed by the ImageJ software bundled with Java 8.

### DLS measurements

DLS data was obtained on a Zetasizer Nano S by Malvern Panalytical. Each measurement was performed three times. A 40 μL plastic cuvette with a stopper was used for each measurement. The measurement was optimized automatically by the instrument and the attenuation was set at 11. The data was exported as a pdf.

### SEM imaging

A JSM-640LV (LVSEM) was utilized to collect scanning electron microscopy (SEM) images at 30 kV.

### XRD analysis

The Powder XRD data was collected on a Bruker D8 Advance A25 system with a monochromated Cu Kα radiation source. The sample was measured under ambient conditions, and the X-ray source was operated at 40 kV and 40 mA.

### Human PBMC preparation and cultures

The study was approved by Colorado Medical Institutional Review Board (COMIRB) and abides by the Declaration of Helsinki principles. Venous blood from healthy consenting donors was drawn into lithium heparin containing tubes and PBMCs were isolated using centrifugation over Ficoll-Hypaque cushions as previously described (62, 76–78). Cells were washed three times with saline and resuspended in RPMI at 5 x 10^6/^mL. For MNPLs stimulation, 0.5 x 10^6^ cells were seeded per well in 96-flat bottom well plates and cultured in a total of 200 μL RPMI with 10% FBS for one to three days, with or without the presence of different concentrations of MNPLs. Aliquots of the MNPLs were freshly diluted in warm RPMI to different concentrations for experiments. After incubation times were completed, supernatants were collected by centrifugation at 400 x g for five minutes and stored at -80°C for further analysis.

### Human whole blood culture

As previously described (62), one mL of heparinized blood was added per well in 24-well plates with or without the presence of 1 mg/mL commercial MNPLs. After three to five days, the supernatants were collected by centrifugation at 400 x g for five minutes and stored at - 80°C for further cytokine analysis.

### Human intestinal epithelial T84 cell culture

For MNPLs stimulation, 80,000 cells were seeded per well in 96-flat bottom well plates and cultured in a total of 200 μL DMEM/F12 (1:1) with 10% FBS for one to five days, with or without the presence of indicated concentrations of MNPLs. Aliquots of the MNPLs were freshly diluted in warm medium to the desired concentrations for experiments. After incubation times were completed, supernatants were collected by centrifugation at 400 x g for five minutes and stored at - 80°C for further analysis.

### Human monocytic THP-1 culture

For MNPLs stimulation, 100,000 cells were seeded per well in 96-round bottom well plates and cultured in a total of 200 μL RPMI with 10% FBS for one to five days, with or without the presence of indicated concentrations of MNPLs. Aliquots of the MNPLs were freshly diluted in warm medium to the desired concentrations for experiments. For Polymyxin B blocking assays, the cells were pretreated with 10 μg/mL Polymyxin B for one hour before they were treated with MNPLs for three to five days. After incubation times were completed, supernatants were collected by centrifugation at 400 x g for five minutes and stored at - 80°C for further analysis.

### Cell morphology and light microscopy

To monitor cell morphology, the human THP-1 or T84 cells or PBMCs were cultured on 96-flat bottom well plates in the cell density as described above. The cells were treated with or without the presence of 1 mg/mL MNPLs for three to five days. The cells in the 96-well plates were viewed under an AmScope IN300TB inverted phase-contrast microscope (AmScope, Irvine, CA). The phase-contrast images were taken with an AmScope microscope digital camera MU1000 using the software AmLite (AmScope).

### Cell death and flow cytometry

For flow cytometry study, 1,000,000 human THP-1 cells were seeded per well in 24-flat bottom well plates and cultured in a total of 2 mL RPMI with 10% FBS. The cells were treated with or without 1 mg/mL MNPLs for five days. After incubation times were completed, the cells were briefly spun down and washed once with ice-cold flow cytometry staining buffer. The cells were then stained as described earlier for flow cytometry (78, 79). Briefly, for non-permeabilized cell surface staining, the cells were stained with FITC-conjugated Annexin V (BioLegend) in Annexin V binding buffer for 15 minutes at RT in the dark and then stained with Propidium Iodide (PI) (Tonbo Biosciences) right before analysis with flow cytometry. The cells were acquired on a BD LSR Fortessa Analyzer (BD Biosciences) and data were analyzed using FlowJo software (Tree Star, Ashland, OR).

### Annexin V staining and fluorescent imaging

Human intestinal epithelial T84 cells were cultured for MNPLs stimulation and imaging. 80,000 cells were seeded per well in 96-flat bottom well plates and cultured in a total of 200 μL DMEM/F12 (1:1) with 10% FBS for five days, with or without the presence of 1mg/mL MNPLs. Aliquots of the MNPLs were freshly diluted in warm medium to the desired concentration for experiments. After incubation times were completed, the cells were washed with PBS, and stained with FITC-conjugated Annexin V (BioLegend) in Annexin V binding buffer for 15 minutes (as described above). The stained cells were washed with PBS and observed under fluorescent microscope. Both fluorescent and phase-contrast images were captured using a Nikon Eclipse Ts2R fluorescent microscope (Nikon Instruments Inc, Melville, NY) with the software NIS-Elements BR5.30.02.

### Statistical analysis

The significance of differences was evaluated with Student’s two-tailed *t* test or one-way ANOVA. The mean or mean fold change for each condition was calculated as indicated. The data shown represent the Mean ± SD.

## Data Availability

The original contributions presented in the study are included in the article/**Supplementary Material**. Further inquiries can be directed to the corresponding authors.

## References

[1] Thompson RC Moore CJ vom Saal FS Swan SH. Plastics, the environment and human health: current consensus and future trends. Philos Trans R Soc London. Ser B Biol Sci. (2009) 364:2153–66. doi: 10.1098/rstb.2009.0053 PMC2873021 19528062

[2] Geyer R Jambeck JR Law KL. Production, use, and fate of all plastics ever made. Sci Adv. (2017) 3. doi: 10.1126/sciadv.1700782 PMC5517107 28776036

[3] Borrelle SB Ringma J Law KL Monnahan CC Lebreton L McGivern A. Predicted growth in plastic waste exceeds efforts to mitigate plastic pollution. Science. (2020) 369:1515–8. doi: 10.1126/science.aba3656 32943526

[4] Yonkos LT Friedel EA Perez-Reyes AC Ghosal S Arthur CD. Microplastics in four estuarine rivers in the chesapeake bay, USA. Environ Sci Technol. (2014) 48:14195–202. doi: 10.1021/es5036317 25389665

[5] Iniguez ME Conesa JA Fullana A. Microplastics in spanish table salt. Sci Rep-Uk. (2017) 7. doi: 10.1038/s41598-017-09128-x PMC5561224 28819264

[6] Leslie HA van Velzen MJM Brandsma SH Vethaak AD Garcia-Vallejo JJ Lamoree MH. Discovery and quantification of plastic particle pollution in human blood. Environ Int. (2022) 163:107199. doi: 10.1016/j.envint.2022.107199 35367073

[7] Yan Z Liu Y Zhang T Zhang F Ren H Zhang Y. Analysis of microplastics in human feces reveals a correlation between fecal microplastics and inflammatory bowel disease status. Environ Sci Technol. (2022) 56:414–21. doi: 10.1021/acs.est.1c03924 34935363

[8] Cox KD Covernton GA Davies HL Dower JF Juanes F Dudas SE. Human consumption of microplastics. Environ Sci Technol. (2019) 53:7068–74. doi: 10.1021/acs.est.9b01517 31184127

[9] Wong J Magun BE Wood LJ. Lung inflammation caused by inhaled toxicants: a review. Int J Chron Obstruct Pulmon Dis. (2016) 11:1391–401. doi: 10.2147/COPD.S106009 PMC4922809 27382275

[10] Calderon-Garciduenas L Franco-Lira M Torres-Jardon R Henriquez-Roldan C Barragan-Mejia G Valencia-Salazar G. Pediatric respiratory and systemic effects of chronic air pollution exposure: nose, lung, heart, and brain pathology. Toxicol Pathol. (2007) 35:154–62. doi: 10.1080/01926230601059985 17325984

[11] Linares R Fernandez MF Gutierrez A Garcia-Villalba R Suarez B Zapater P. Endocrine disruption in Crohn's disease: Bisphenol A enhances systemic inflammatory response in patients with gut barrier translocation of dysbiotic microbiota products. FASEB J. (2021) 35:e21697. doi: 10.1096/fj.202100481R 34085740

[12] Malaise Y Menard S Cartier C Lencina C Sommer C Gaultier E. Consequences of bisphenol a perinatal exposure on immune responses and gut barrier function in mice. Arch Toxicol. (2018) 92:347–58. doi: 10.1007/s00204-017-2038-2 28733891

[13] Santoro A Scafuro M Troisi J Piegari G Di Pietro P Mele E. Multi-systemic alterations by chronic exposure to a low dose of bisphenol A in drinking water: effects on inflammation and NAD(+)-dependent deacetylase sirtuin1 in lactating and weaned rats. Int J Mol Sci. (2021) 22. doi: 10.3390/ijms22189666 PMC8467074 34575829

[14] Gianniou N Katsaounou P Dima E Giannakopoulou CE Kardara M Saltagianni V. Prolonged occupational exposure leads to allergic airway sensitization and chronic airway and systemic inflammation in professional firefighters. Respir Med. (2016) 118:7–14. doi: 10.1016/j.rmed.2016.07.006 27578465

[15] Marzec JM Nadadur SS. Inflammation resolution in environmental pulmonary health and morbidity. Toxicol Appl Pharmacol. (2022) 449:116070. doi: 10.1016/j.taap.2022.116070 35618031 PMC9872158

[16] Wiegman CH Li F Ryffel B Togbe D Chung KF. Oxidative stress in ozone-Induced chronic lung inflammation and emphysema: A facet of chronic obstructive pulmonary disease. Front Immunol. (2020) 11:1957. doi: 10.3389/fimmu.2020.01957 32983127 PMC7492639

[17] Lee KW Shim WJ Kwon OY Kang JH. Size-Dependent effects of micro polystyrene particles in the marine copepod tigriopus japonicus. Environ Sci Technol. (2013) 47:11278–83. doi: 10.1021/es401932b 23988225

[18] Della Torre C Bergami E Salvati A Faleri C Cirino P Dawson KA. Accumulation and Embryotoxicity of Polystyrene Nanoparticles at Early Stage of Development of Sea Urchin Embryos Paracentrotus lividus. Environ Sci Technol. (2014) 48:12302–11. doi: 10.1021/es502569w 25260196

[19] Li B Ding Y Cheng X Sheng D Xu Z Rong Q. Polyethylene microplastics affect the distribution of gut microbiota and inflammation development in mice. Chemosphere. (2020) 244:125492. doi: 10.1016/j.chemosphere.2019.125492 31809927

[20] Merkley SD Moss HC Goodfellow SM Ling CL Meyer-Hagen JL Weaver J. Polystyrene microplastics induce an immunometabolic active state in macrophages. Cell Biol Toxicol. (2022) 38:31–41. doi: 10.1007/s10565-021-09616-x 34021430 PMC8606615

[21] Jenner LC Rotchell JM Bennett RT Cowen M Tentzeris V Sadofsky LR. Detection of microplastics in human lung tissue using muFTIR spectroscopy. Sci Total Environ. (2022) 831:154907. doi: 10.1016/j.scitotenv.2022.154907 35364151

[22] Zhu L Kang Y Ma M Wu Z Zhang L Hu R. Tissue accumulation of microplastics and potential health risks in human. Sci Total Environ. (2024) 915:170004. doi: 10.1016/j.scitotenv.2024.170004 38220018

[23] Pironti C Notarstefano V Ricciardi M Motta O Giorgini E Montano L. First evidence of microplastics in human urine, a preliminary study of intake in the human body. Toxics. (2022) 11. doi: 10.3390/toxics11010040 PMC9867291 36668766

[24] Schwabl P Koppel S Konigshofer P Bucsics T Trauner M Reiberger T. Detection of various microplastics in human stool: A prospective case series. Ann Intern Med. (2019) 171:453–7. doi: 10.7326/M19-0618 31476765

[25] Horvatits T Tamminga M Liu B Sebode M Carambia A Fischer L. Microplastics detected in cirrhotic liver tissue. EBioMedicine. (2022) 82:104147. doi: 10.1016/j.ebiom.2022.104147 35835713 PMC9386716

[26] Ragusa A Svelato A Santacroce C Catalano P Notarstefano V Carnevali O. Plasticenta: First evidence of microplastics in human placenta. Environ Int. (2021) 146:106274. doi: 10.1016/j.envint.2020.106274 33395930

[27] Marfella R Prattichizzo F Sardu C Fulgenzi G Graciotti L Spadoni T. Microplastics and nanoplastics in atheromas and cardiovascular events. N Engl J Med. (2024) 390:900–10. doi: 10.1056/NEJMoa2309822 PMC11009876 38446676

[28] Rubin AE Zucker I. Interactions of microplastics and organic compounds in aquatic environments: A case study of augmented joint toxicity. Chemosphere. (2022) 289:133212. doi: 10.1016/j.chemosphere.2021.133212 34890605

[29] Andrew J Whelton AS Isaacson KP. Plastic pipes are polluting drinking water systems after wildfires – it’s a risk in urban fires, too. Conversation. (2020).

[30] Isaacson KP Proctor CR Wang QE Edwards EY Noh Y Shah AD. Drinking water contamination from the thermal degradation of plastics: implications for wildfire and structure fire response. Environ Science: Water Res Technol. (2021) 7:274–84. doi: 10.1039/D0EW00836B

[31] Liu S Shi J Wang J Dai Y Li H Li J. Interactions between microplastics and heavy metals in aquatic environments: A review. Front Microbiol. (2021) 12:652520. doi: 10.3389/fmicb.2021.652520 33967988 PMC8100347

[32] Menéndez-Pedriza A Jaumot J. Interaction of environmental pollutants with microplastics: A critical review of sorption factors, bioaccumulation and ecotoxicological effects. Toxics. (2020) 8:40. doi: 10.3390/toxics8020040 32498316 PMC7355763

[33] Robin M. How wildfires can contaminate drinking water. Chem Eng News. (2022) 100.

[34] Lohmann R. Microplastics are not important for the cycling and bioaccumulation of organic pollutants in the oceans-but should microplastics be considered POPs themselves? Integr Environ Assess Manag. (2017) 13:460–5. doi: 10.1002/ieam.1914 28440937

[35] Shams M Alam I Chowdhury I. Aggregation and stability of nanoscale plastics in aquatic environment. Water Res. (2020) 171:115401. doi: 10.1016/j.watres.2019.115401 31884379

[36] Li S Liu H Gao R Abdurahman A Dai J Zeng F. Aggregation kinetics of microplastics in aquatic environment: Complex roles of electrolytes, pH, and natural organic matter. Environ pollut. (2018) 237:126–32. doi: 10.1016/j.envpol.2018.02.042 29482018

[37] Stetefeld J McKenna SA Patel TR. Dynamic light scattering: a practical guide and applications in biomedical sciences. Biophys Rev. (2016) 8:409–27. doi: 10.1007/s12551-016-0218-6 PMC5425802 28510011

[38] Smith BC. The infrared spectra of polymers II: polyethylene. Spectroscopy. (2021) 36:24–9. doi: 10.56530/spectroscopy

[39] Smith BC. The infrared spectra of polymers III: hydrocarbon polymers. Spectroscopy. (2021) 36:22–5. doi: 10.56530/spectroscopy

[40] Smith BC. The infrared spectra of polymers VIII: polyesters and the rule of three. Spectroscopy. (2022) 37:25–8. doi: 10.56530/spectroscopy.ta9383e3

[41] Sancataldo G Ferrara V Bonomo FP Chillura Martino DF Licciardi M Pignataro BG. Identification of microplastics using 4-dimethylamino-4'-nitrostilbene solvatochromic fluorescence. Microsc Res Tech. (2021) 84:2820–31. doi: 10.1002/jemt.v84.12 PMC9291063 34047435

[42] Ho D Liu S Wei H Karthikeyan KG. The glowing potential of Nile red for microplastics Identification: Science and mechanism of fluorescence staining. Microchemical J. (2023) 197:109708. doi: 10.1016/j.microc.2023.109708

[43] Ribeiro F Duarte AC da Costa JP. Staining methodologies for microplastics screening. TrAC Trends Analytical Chem. (2024) 172:117555. doi: 10.1016/j.trac.2024.117555

[44] Karakolis E Nguyen B You J Rochman C Sinton D. Fluorescent dyes for visualizing microplastic particles and fibers in laboratory-based studies. Environ Sci Technol Lett. (2019) 6:334–340. doi: 10.1021/acs.estlett.9b00241

[45] Gao Z Wontor K Cizdziel JV. Labeling microplastics with fluorescent dyes for detection, recovery, and degradation experiments. Molecules. (2022) 27. doi: 10.3390/molecules27217415 PMC9653731 36364240

[46] Manikandan N. XRD, FTIR and the optical studies of pure polystyrene film. Int J Recent Innov Trends Comput Commun. (2014) 2:1148–51.

[47] Benetti EM Causin V Marega C Marigo A Ferrara G Ferraro A. Morphological and structural characterization of polypropylene based nanocomposites. Polymer. (2005) 46:8275–85. doi: 10.1016/j.polymer.2005.06.056

[48] Singh D Malik H Gupta C Singh V. X-ray diffraction studies for identification of polyethylene terephthalate fibres. Indian J Sci Technol. (2017) 10:1–4. doi: 10.17485/ijst/2017/v10i17/110232

[49] Kawaguchi T Ito T Kawai H Keedy D Stein R. Dynamic X-ray diffraction from polyethylene. Macromolecules. (1968) 1:126–33. doi: 10.1021/ma60002a005

[50] Schrank I Möller JN Imhof HK Hauenstein O Zielke F Agarwal S. Microplastic sample purification methods - Assessing detrimental effects of purification procedures on specific plastic types. Sci Total Environ. (2022) 833:154824. doi: 10.1016/j.scitotenv.2022.154824 35351498

[51] Hu K Zhou P Yang Y Hall T Nie G Yao Y. Degradation of microplastics by a thermal fenton reaction. ACS ES&T Eng. (2022) 2:110–20. doi: 10.1021/acsestengg.1c00323

[52] Kovačić M Tomić A Tonković S Pulitika A Papac Zjačić J Katančić Z. Pristine and UV-weathered PET microplastics as water contaminants: appraising the potential of the fenton process for effective remediation. Processes. (2024) 12:844. doi: 10.3390/pr12040844

[53] Bule Možar K Miloloža M Martinjak V Radovanović-Perić F Bafti A Ujević Bošnjak M. Evaluation of fenton, photo-Fenton and fenton-like processes in degradation of PE, PP, and PVC microplastics. Water. (2024) 16:673. doi: 10.3390/w16050673

[54] Meides N Menzel T Poetzschner B Löder MGJ Mansfeld U Strohriegl P. Reconstructing the environmental degradation of polystyrene by accelerated weathering. Environ Sci Technol. (2021) 55:7930–8. doi: 10.1021/acs.est.0c07718 34018732

[55] Da Costa J Nunes A Santos P Girão A Duarte A Rocha-Santos T. Degradation of polyethylene microplastics in seawater: Insights into the environmental degradation of polymers. J Environ Sci Health Part A. (2018) 53:1–10. doi: 10.1080/10934529.2018.1455381 29624466

[56] Rana AK Thakur MK Saini AK Mokhta SK Moradi O Rydzkowski T. Recent developments in microbial degradation of polypropylene: Integrated approaches towards a sustainable environment. Sci Total Environ. (2022) 826:154056. doi: 10.1016/j.scitotenv.2022.154056 35231525

[57] Dinarello CA. Anti-inflammatory agents: present and future. Cell. (2010) 140:935–50. doi: 10.1016/j.cell.2010.02.043 PMC3752337 20303881

[58] Mantovani A Dinarello CA Molgora M Garlanda C. Interleukin-1 and related cytokines in the regulation of inflammation and immunity. Immunity. (2019) 50:778–95. doi: 10.1016/j.immuni.2019.03.012 PMC7174020 30995499

[59] Kimbrell DA Beutler B. The evolution and genetics of innate immunity. Nat Rev Genet. (2001) 2:256–67. doi: 10.1038/35066006 11283698

[60] Netea MG Azam T Ferwerda G Girardin SE Walsh M Park JS. IL-32 synergizes with nucleotide oligomerization domain (NOD) 1 and NOD2 ligands for IL-1b and IL-6 production through a caspase 1-dependent mechanism. Proc Natl Acad Sci U.S.A. (2005) 102:16309–14. doi: 10.1073/pnas.0508237102 PMC1283464 16260731

[61] Nold MF Nold-Petry CA Zepp JA Palmer BE Bufler P Dinarello CA. IL-37 is a fundamental inhibitor of innate immunity. Nat Immunol. (2010) 11:1014–22. doi: 10.1038/ni.1944 PMC3537119 20935647

[62] Li S Jiang L Beckmann K Hojen JF Pessara U Powers NE. A novel anti-human IL-1R7 antibody reduces IL-18-mediated inflammatory signaling. J Biol Chem. (2021) 296:100630. doi: 10.1016/j.jbc.2021.100630 33823154 PMC8018910

[63] Molmenti EP Ziambaras T Perlmutter DH. Evidence for an acute phase response in human intestinal epithelial cells. J Biol Chem. (1993) 268:14116–24. doi: 10.1016/S0021-9258(19)85216-X 7686149

[64] Cole M Webb H Lindeque PK Fileman ES Halsband C Galloway TS. Isolation of microplastics in biota-rich seawater samples and marine organisms. Sci Rep. (2014) 4:4528. doi: 10.1038/srep04528 24681661 PMC3970126

[65] Fischer EK Paglialonga L Czech E Tamminga M. Microplastic pollution in lakes and lake shoreline sediments - A case study on Lake Bolsena and Lake Chiusi (central Italy). Environ pollut. (2016) 213:648–57. doi: 10.1016/j.envpol.2016.03.012 27104923

[66] Zhao S Zhu L Li D. Microplastic in three urban estuaries, China. Environ pollut. (2015) 206:597–604. doi: 10.1016/j.envpol.2015.08.027 26312741

[67] Ronca S. Chapter 10 - Polyethylene. In: Gilbert M, editor. Brydson's Plastics Materials, Eighth Edition. Butterworth-Heinemann: Elsevier Ltd. (2017). p. 247–78.

[68] Fu D Zhang Q Fan Z Qi H Wang Z Peng L. Aged microplastics polyvinyl chloride interact with copper and cause oxidative stress towards microalgae Chlorella vulgaris. Aquat Toxicol. (2019) 216:105319. doi: 10.1016/j.aquatox.2019.105319 31586885

[69] Davarpanah E Guilhermino L. Single and combined effects of microplastics and copper on the population growth of the marine microalgae Tetraselmis chuii. Estuar Coast Shelf Sci. (2015) 167:269–75. doi: 10.1016/j.ecss.2015.07.023

[70] Xie S Zhang R Li Z Liu C Chen Y Yu Q. Microplastics perturb colonic epithelial homeostasis associated with intestinal overproliferation, exacerbating the severity of colitis. Environ Res. (2023) 217:114861. doi: 10.1016/j.envres.2022.114861 36410465

[71] Chen Y Williams AM Gordon EB Rudolph SE Longo BN Li G. Biological effects of polystyrene micro- and nano-plastics on human intestinal organoid-derived epithelial tissue models without and with M cells. Nanomedicine. (2023) 50:102680. doi: 10.1016/j.nano.2023.102680 37105344 PMC10247512

[72] Qian N Gao X Lang X Deng H Bratu TM Chen Q. Rapid single-particle chemical imaging of nanoplastics by SRS microscopy. Proc Natl Acad Sci U.S.A. (2024) 121:e2300582121. doi: 10.1073/pnas.2300582121 38190543 PMC10801917

[73] (NOAA). Laboratory Methods for the Analysis of Microplastics in the Marine Environment: Recommendations for quantifying synthetic particles in waters and sediments. Tech Memorandum NOS-OR&R-48. (2015), 3–12.

[74] Lin Y Zhang Y Wang Y Lv Y Yang L Chen Z. Efficient degradation and mineralization of polyethylene terephthalate microplastics by the synergy of sulfate and hydroxyl radicals in a heterogeneous electro-Fenton-activated persulfate oxidation system. J Hazardous Materials. (2024) 478:135635. doi: 10.1016/j.jhazmat.2024.135635 39182298

[75] Duong TT Le PT Nguyen TNH Hoang TQ Ngo HM Doan TO. Selection of a density separation solution to study microplastics in tropical riverine sediment. Environ Monit Assess. (2022) 194:65. doi: 10.1007/s10661-021-09664-0 34993616

[76] Leoni F Fossati G Lewis EC Lee JK Porro G Pagani P. The histone deacetylase inhibitor ITF2357 reduces production of pro-inflammatory cytokines *in vitro* and systemic inflammation in *vivo* . Mol Med. (2005) 11:1–15. doi: 10.2119/2006-00005.Dinarello 16557334 PMC1449516

[77] Li S Fossati G Marchetti C Modena D Pozzi P Reznikov LL. Specific inhibition of histone deacetylase 8 reduces gene expression and production of proinflammatory cytokines *in vitro* and in *vivo* . J Biol Chem. (2015) 290:2368–78. doi: 10.1074/jbc.M114.618454 PMC4303687 25451941

[78] Li S Neff CP Barber K Hong J Luo Y Azam T. Extracellular forms of IL-37 inhibit innate inflammation *in vitro* and *in vivo* but require the IL-1 family decoy receptor IL-1R8. Proc Natl Acad Sci U.S.A. (2015) 112:2497–502. doi: 10.1073/pnas.1424626112 PMC4345608 25654981

[79] Li S Amo-Aparicio J Neff CP Tengesdal IW Azam T Palmer BE. Role for nuclear interleukin-37 in the suppression of innate immunity. Proc Natl Acad Sci U.S.A. (2019) 116:4456–61. doi: 10.1073/pnas.1821111116 PMC6410848 30792349

