# Supplementary material for: Micro- and nano-plastics induce inflammation and cell death in human cells
Source: Front Immunol. 2025 Mar 31;16:1528502. doi: 10.3389/fimmu.2025.1528502 (PMC11995046; doi:10.3389/fimmu.2025.1528502)
Supplement: Supplementary file 1 [file DataSheet1.pdf]

SUPPORTING INFORMATION

Micro- and Nano-plastics Induce Inflammation and Cell Death in Human Cells

Brandon Bishop[#,1], William S. Webber[#,2], Shaikh M. Atif[3], Ashley Ley[1], Karl A. Pankratz[2], Rachael Kostelecky[4], Sean P. Colgan[4], Charles A. Dinarello[2], Wei Zhang[*,1] and Suzhao Li[*,2]

[1]Department of Chemistry, University of Colorado Boulder, Boulder, CO 80309, USA
[2]Department of Medicine, University of Colorado Denver Anschutz Medical Campus, Aurora, CO 80045, USA
[3]Division of Allergy and Clinical Immunology, Department of Medicine, University of Colorado Denver Anschutz Medical Campus, Aurora, CO 80045, USA
[4] Mucosal Inflammation Program, Division of Gastroenterology and Hepatology, Department of Medicine, University of Colorado Anschutz Medical Campus, Aurora, CO 80045, USA

[#]These authors contributed equally to this work.

*To whom correspondence should be addressed: Wei Zhang, Department of Chemistry, University of Colorado Boulder; and Suzhao Li, Department of Medicine, University of Colorado Denver Anschutz Medical Campus, Aurora, Colorado 80045, USA

Email: wei.zhang@colorado.edu; suzhao.li@cuanschutz.edu

Keywords: micro- and nano- plastics (MNPLs) inflammation cell death human environment health

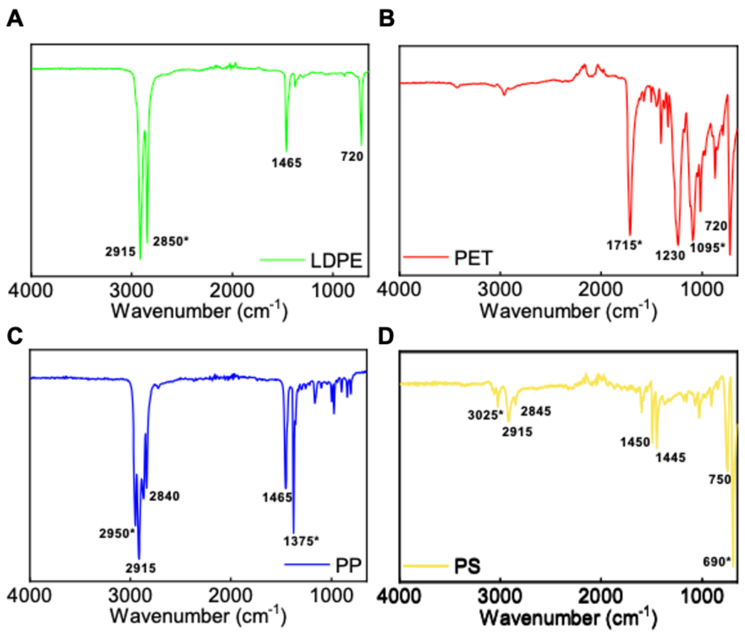

Supplemental Figure 1. Fourier transform infrared spectroscopy (FTIR) spectra of standard MNPLs obtained from commercial sources. The major peaks have been labeled with their respective wavenumbers and the peaks used for comparison to the environmental samples have been additionally labeled with an asterisk. The color of the spectrum corresponds to the color after staining. Plastic, color, peak used for comparison: **(A)** Polyethylene (PE), green, 720 cm^{-1} and 2850 cm^{-1}, **(B)** Polyethylene terephthalate (PET), red, 1715 cm^{-1} and 1095 cm^{-1}, **(C)** Polypropylene (PP), blue, 2950 cm^{-1} and 1375 cm^{-1}, **(D)** Polystyrene (PS), yellow, 690 cm^{-1} and 3025 cm^{-1}.

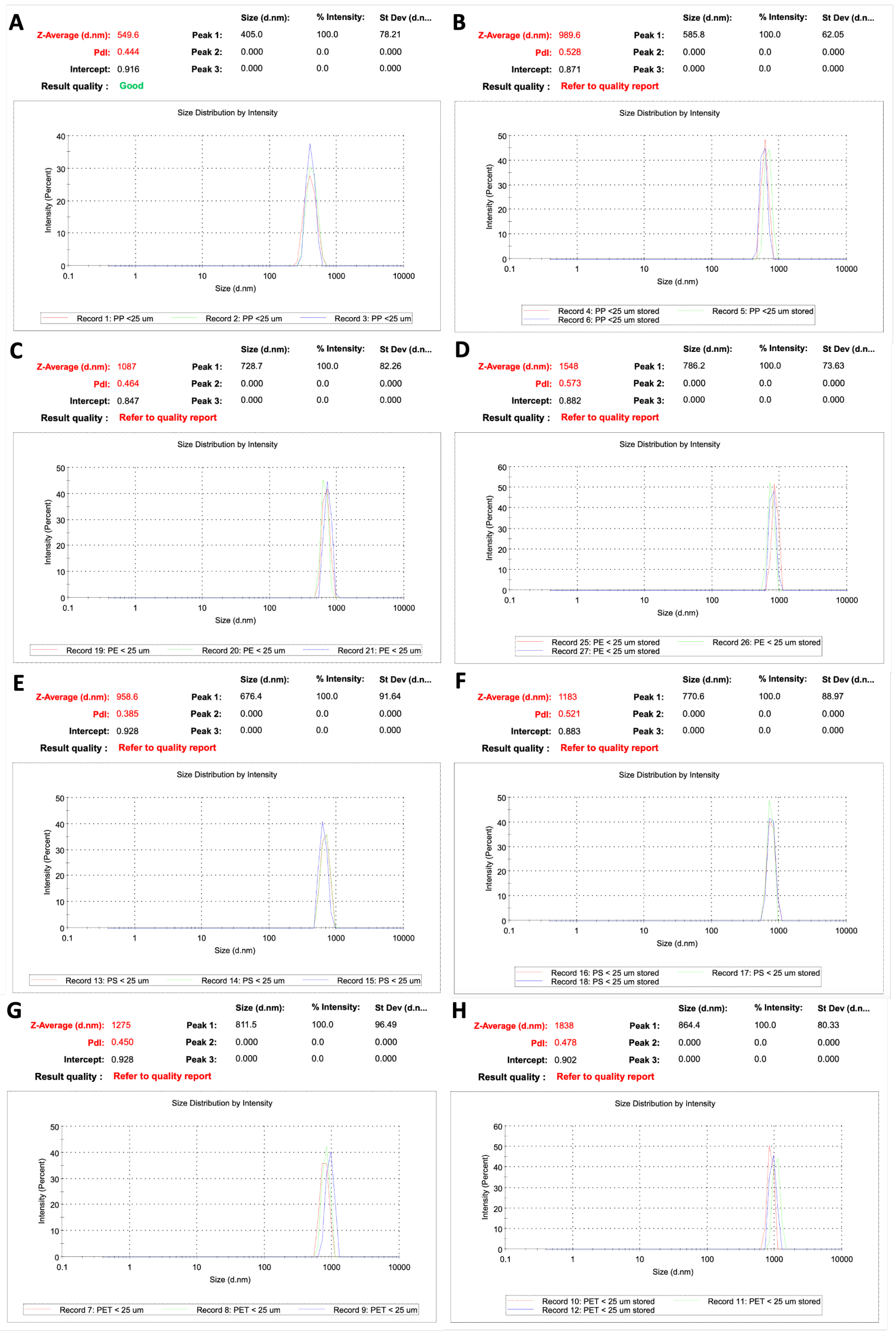

Supplemental Figure 2. Dynamic light scattering (DLS) measurements for the four commercial polymer solutions containing MNPLs < 0.4 µm. (A) DLS of PP MNPLs < 0.4 µm after filtration and **(B)** after being stored. **(C)** DLS of PE MNPLs < 0.4 µm after filtration and **(D)** after being stored. **(E)** DLS of PS MNPLs < 0.4 µm after filtration and **(F)** after being stored. **(G)** DLS of PET MNPLs < 0.4 µm after filtration and **(H)** after being stored.

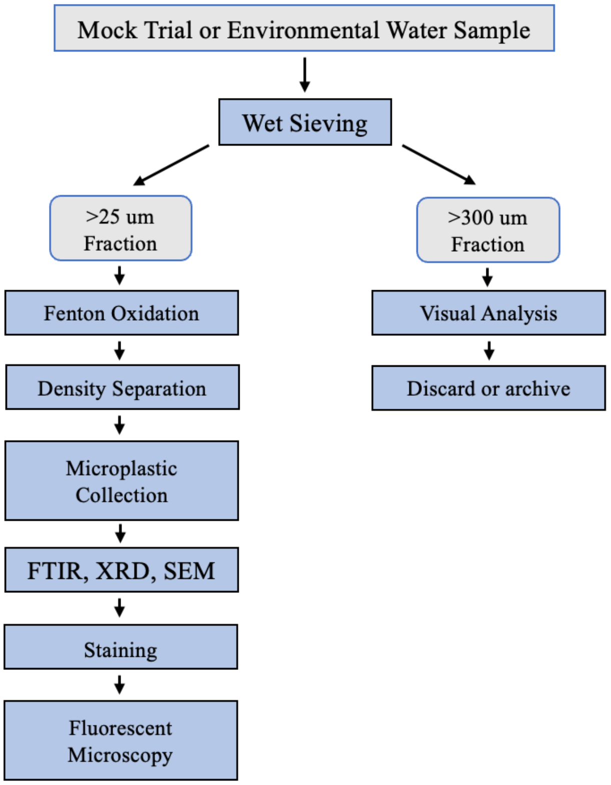

Supplemental Figure 3. Schematic of procedure used to obtain and analyze the microplastics from mock trial and Greater Denver area water sources. The staining of the obtained plastics was done using 4-dimethylamino-4'-nitrostilbene (DANS) fluorescent dye following a reported procedure [(1)].

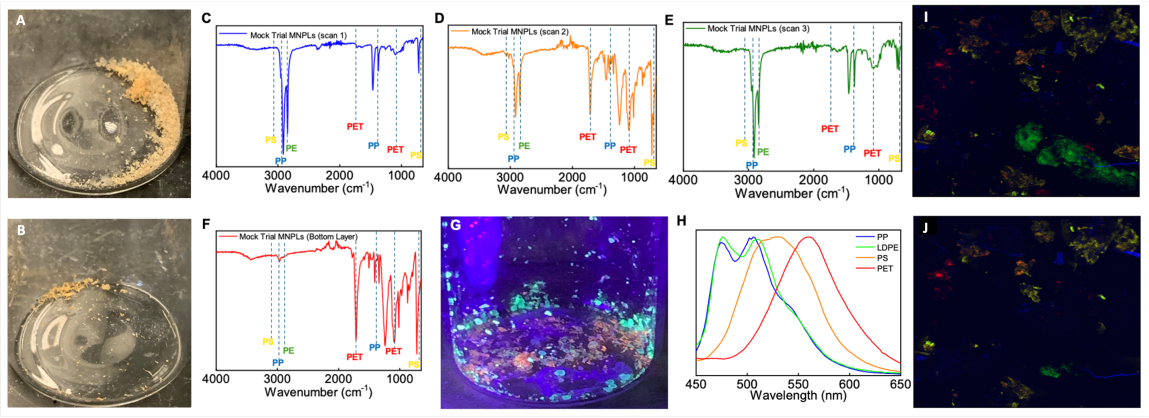

Supplemental Figure 4. Plastic composition analysis of mock trial. (A) MPs collected from the top layer of the density separation performed during the mock trial completed using commercial standards. (**B)** MPs collected from the bottom layer of the density separation (inorganic sediment layer) performed during the mock trial completed using commercial standards. (**C-F)** FTIR spectra of MPs collected from the density separation performed during the mock trial. The major peaks corresponding to the four standard plastics have been labeled for each of the FTIR spectra. **(C-E)** FTIR spectra of MPs collected from the top layer of the extraction obtained from the mock trial. Multiple scans (Scans 1-3) were necessary to identify all the plastics due to the amount of material obtained (20 mg). (**F)** An FTIR spectrum of the bottom layer of the density separation. PET (1715 cm^{-1}) appears to be the only plastic lost in a substantial amount. (**G)** Image of stained MPs from the mock trial performed, when illuminated by UV LED. **(H)** Normalized steady-state fluorescence spectrums obtained from the mixture of MPs obtained from the mock trial, showing that the individual spectra of each plastic can be separated in a mixture. **(I-J)** Fluorescent confocal microscopy images of the plastics obtained from the mock trial.

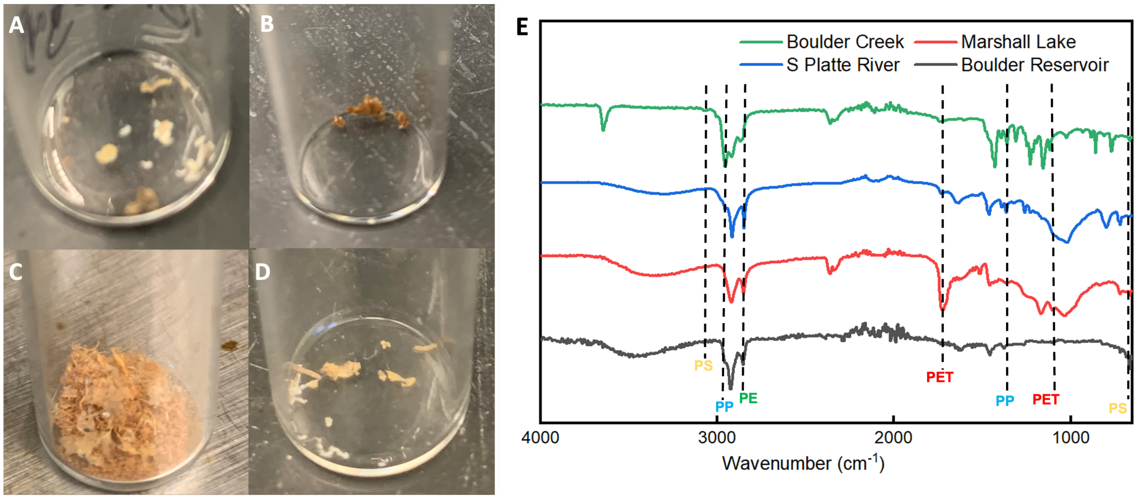

Supplemental Figure 5. Images of the environmental MPs obtained and the corresponding FTIR Spectra for each environmental sample. (A-D) MPs collected from multiple water sources surrounding the Greater Denver area. (**A)** MPs collected from Boulder Creek. (**B)** MPs collected from Boulder Reservoir. (**C)** MPs collected from Marshall Lake. (**D)** MPs collected from South Platte River (S Platte River). (**E)** FTIR spectra of MPs obtained from multiple water sources surrounding the Greater Denver area. The major peaks used to identify standard plastics have been labeled with the plastics they correspond to.

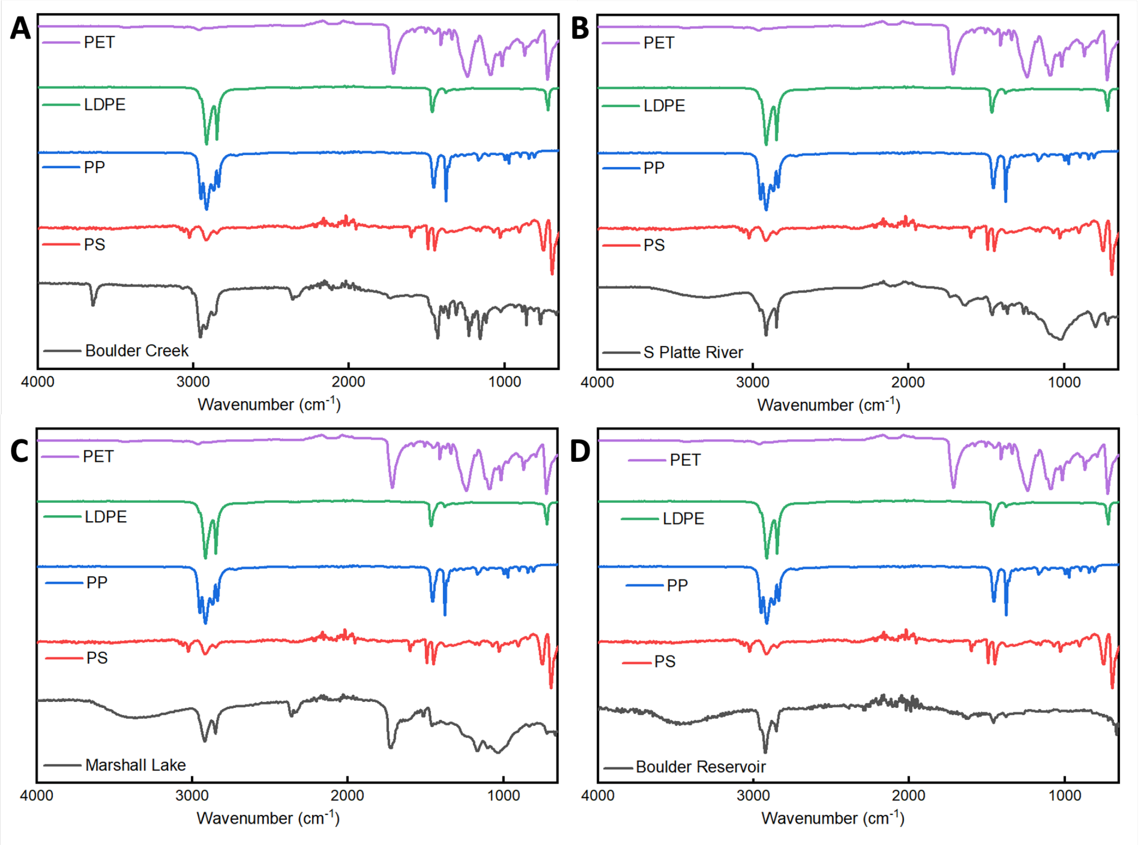

Supplemental Figure 6. FTIR spectra for each environmental location compared to the FTIR spectra of each commercial polymer. (A) FTIR of MPs collected from Boulder Creek compared to the four commercial plastics. **(B)** FTIR MPs collected from South Platte River compared to the four commercial plastics. **(C)** FTIR MPs collected from Marshall Lake compared to the four commercial plastics. **(D)** FTIR MPs collected from Boulder Reservoir compared to the four commercial plastics.

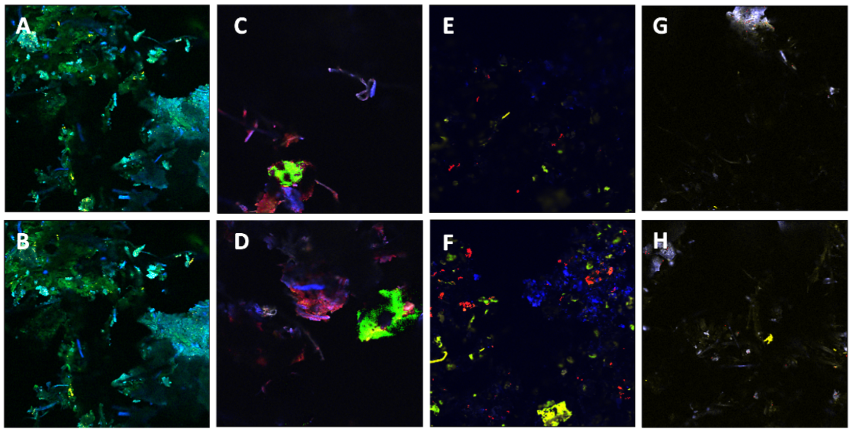

Supplemental Figure 7. Additional fluorescent confocal microscopy images of the environmental MPs obtained from local water sources surrounding the Greater Denver area. (**A-B)** North Boulder Creek. (**C-D)** South Platte River. (**E-F)** Marshall Lake. (**G-H)** Boulder Reservoir.

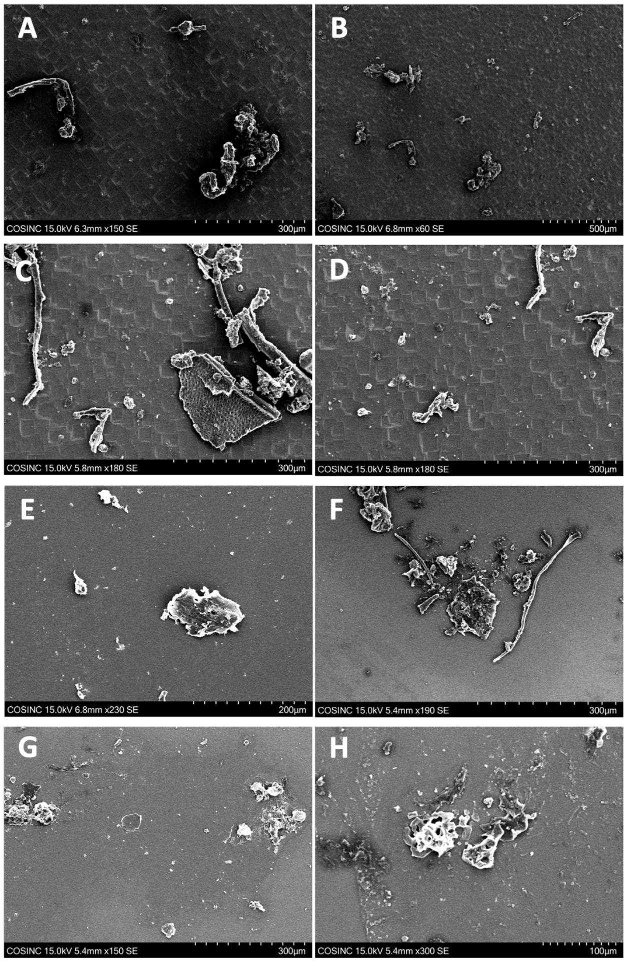

Supplemental Figure 8. Scanning electron microscopy (SEM) images of the environmental MPs obtained. **(A-B)** Boulder Creek. **(C-D)** Boulder Reservoir. **(E-F)** South Platte River. **(G-H)** Marshall Lake.

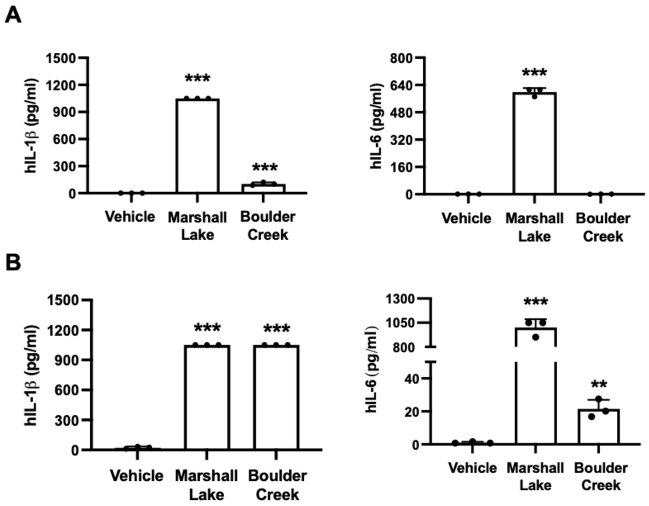

Supplemental Figure 9. Prolonged exposure increased the inflammatory effects of MPs from natural water sources in human cells. (A) One-day and **(B)** Five-day effects of MPs from Marshall Lake and Boulder Creek water samples on inflammatory cytokine production in human THP-1 cells. Human THP-1 cells were treated with or without 1 mg/mL MPs from natural water sources for one to five days. N = 3, Mean ± SD. ** $P < 0.01$, *** $P < 0.001$ compared to vehicle control.

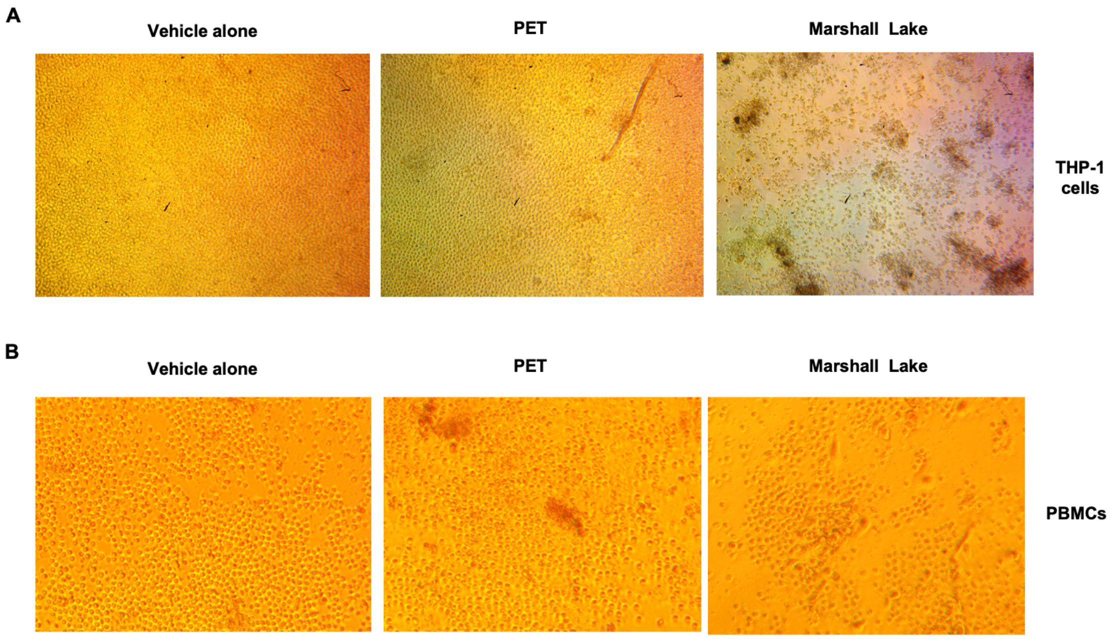

Supplemental Figure 10. The effects of MNPLs of PET origin on human cell morphology. (A) Phase-contrast images of human THP-1 cells treated with or without 1mg/mL commercial MNPLs of PET origin or environmental MPs from Marshall Lake rich in PET for five days. **(B)** Phase-contrast images of human peripheral blood mononuclear cells (PBMCs) treated with or without 1mg/mL commercial MNPLs of PET origin or environmental MPs from Marshall Lake rich in PET for five days. Representative images of at least two independent experiments.

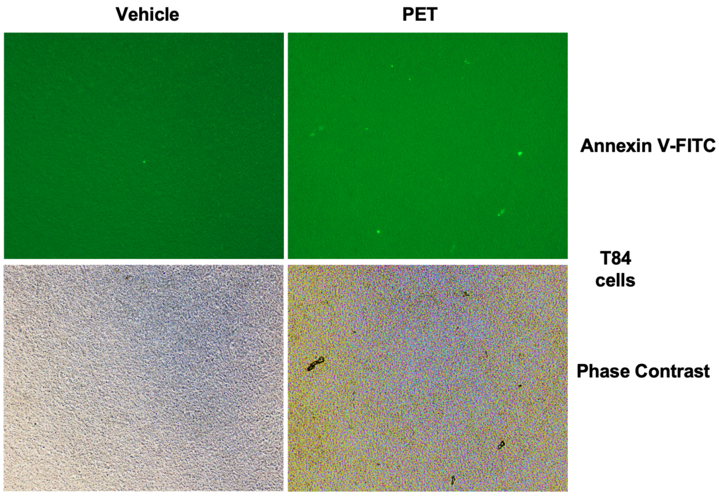

Supplemental Figure 11. Impacts of commercial MNPLs of PET origin on the cell apoptosis and cell morphology of human epithelial T84 cells. Human T84 cells were treated with or without 1 mg/mL commercial MNPLs of PET origin for five days. The T84 cells were stained with FITC-labeled Annexin V and imaged under fluorescent microscope. Both fluorescent and phase-contrast images were captured for presentation purposes. Representative images of two independent experiments.

1. G. Sancataldo *et al.*, Identification of microplastics using 4-dimethylamino-4'-nitrostilbene solvatochromic fluorescence. *Microsc Res Tech* **84**, 2820-2831 (2021).
